# Cork oak aquaporins: functional diversity and regulation with insights into drought response

**DOI:** 10.1007/s00299-026-03869-8

**Published:** 2026-06-06

**Authors:** Farzana Sabir, Raquel Quaresma, Sérgio Paulino, Luísa C. Carvalho, Graça Soveral, Leonor Morais-Cecílio, Catarina Prista

**Affiliations:** 1 LEAF—Linking Landscape, Environment, Agriculture and Food Research Center, Associate Laboratory TERRA, Instituto Superior de Agronomia, University of Lisbon, Tapada da Ajuda, 1349-017 Lisbon, Portugal; 2 Research Institute for Medicines (iMed.ULisboa), Faculty of Pharmacy, Universidade de Lisboa, 1649-003 Lisbon, Portugal

**Keywords:** *Quercus suber* L., Water homeostasis, Water channels, Stopped-flow spectroscopy, Immunolocalization, Water stress

## Abstract

**Key message:**

Cork oak aquaporins exhibit diverse functional roles in water and solute transport, coordinating tissue-specific and drought-responsive mechanisms that regulate plant water balance and enhance adaptation to increasing climate-driven water scarcity.

**Abstract:**

Cork oak woodlands are increasingly exposed to water scarcity due to climate change. At the cellular level, aquaporins (AQPs) regulate water and solute transport, yet their roles in cork oak remain largely unexplored. To bridge this knowledge gap, we examined the functional diversity, tissue localization, and drought response of cork oak AQPs and their contribution to plant water relations under stress. Four cork oak AQPs, QsPIP2;4, QsTIP2;1, QsNIP1;2, and QsNIP6;1, were expressed in *Saccharomyces cerevisiae* to characterize their functional properties. Stopped-flow spectroscopy showed that QsPIP2;4, QsTIP2;1, and QsNIP1;2 transport water, while QsNIPs facilitate glycerol permeability. Mercury chloride unexpectedly activated QsPIP2;4, an effect abolished in the C69A mutant, highlighting the role of Cys-69 in mercury binding and channel regulation. Yeast growth assays showed that QsNIP6;1 transports boron, hydrogen peroxide, and arsenic, suggesting broader physiological functions of cork AQPs. At the tissue level, immunolocalization showed widespread AQPs’ accumulation across multiple tissue layers of young stems. Higher PIP2s abundance suggests they are likely the main contributors to water transport in these tissues. *QsAQPs* expression under mild drought stress showed that *PIP* and *TIP* were upregulated in leaves and stems, supporting their role in water homeostasis, whereas their downregulation in roots suggests a strategy to restrict water loss back to drying soils. Together, these results provide the first comprehensive molecular, functional, and physiological characterization of cork oak AQPs. They demonstrate how distinct AQPs coordinate water and solute transport under drought, advancing the understanding of cork oak water relations under extreme climatic conditions.

**Supplementary Information:**

The online version contains supplementary material available at 10.1007/s00299-026-03869-8.

## Introduction

Cork oak (*Quercus suber* L.) is a widely distributed species across the Mediterranean basin. In the southern Iberian Peninsula, this special ecosystem is known as *montado* in Portugal and *dehesa* in Spain (Pinto-Correia et al. 2011). The biggest and best representation of *montado* is distributed in the southern half of Portugal. This ecosystem provides a high level of biodiversity and conservation of several endemic and protected species of flora and fauna (Sá-Sousa 2014). Cork oak is protected by Portuguese law and was designated Portugal’s National Tree due to its cultural, ecological, and economic value (Lopes et al. 2019). Cork is the most important forest product from *montado* systems in Portugal. Portuguese cork represents 50% of the world’s cork (Sá-Sousa 2014). However, the persistence of these important forests over the coming decades is uncertain due to various abiotic and biotic factors, as well as human activities (Ibáñez et al. 2017; Kim et al. 2017; Lopes-Fernandes et al. 2024). Numerous studies have suggested the hydraulic failure induced by water scarcity as a primary mechanism responsible for widespread oak decline (Costa et al. 2010; Camilo-Alves et al. 2017; Kim et al. 2017; Rodríguez-Calcerrada et al. 2017; Leite et al. 2020). Low cellular water availability triggers other detrimental scenarios like nutrient imbalance, reduced carbon assimilation, and eventually vulnerability to pathogens, which is another major factor in high oak tree mortality (Sebastiana et al. 2018). Besides, accumulated toxic metal(loids) like arsenic, and other potentially hazardous elements (PHE) in the soil, mainly due to mining activities (Pratas et al. 2013), also represent a threat to the forest system. Therefore, to understand and mitigate the consequences of major constraints on the cork forest system, it is necessary to comprehend the molecular mechanisms regulating cellular water entry and the efficient translocation of nutrients and PHE.

In higher plants, aquaporins (AQPs) facilitate water, nutrients, and metalloid transport. They belong to the membrane intrinsic protein (MIP) family (Maurel and Chrispeels 2001). AQPs enable quick and reversible changes in cellular hydraulic conductance, creating a strong relationship between water transport and plant growth and environmental adaptation. Plant genomes have many folds more AQPs genes than other organisms, implying their unique necessity for growth and adaptation under environmental stress conditions (Maurel et al. 2015; Fox et al. 2017). Plant AQPs are classified into five main subfamilies: 1) PIPs-Plasma membrane Intrinsic Proteins, 2) TIPs -Tonoplast Intrinsic Proteins, 3) SIPs-Small basic Intrinsic Proteins, 4) NIPs-Nodulin 26-like Intrinsic Proteins, 5) XIPs-Uncharacterized Intrinsic Protein. Each subfamily has a unique functional role in plant water movement, nutrient absorption and environmental stress adaptation. PIPs are primarily associated with water transport across plasma membranes, while TIPs help in maintaining the osmotic balance within vacuoles (Maurel et al. 2015). NIPs, SIPs, and XIPs are known for their specialized functions, including the transport of glycerol, metalloids, and defense-related molecules like boron, ammonia, urea, glycerol, selenium, arsenic, and gases like CO_2_ and reactive oxygen species (ROS) such as H_2_O_2_ (Hove and Bhave 2011; Bienert and Bienert 2017). Two highly conserved Asn-Pro-Ala (NPA) motifs form the charge selectivity filter of the channel, preventing charged molecules from permeating the aqueous pore, whereas the ar/R (aromatic/Arg) constrictions present at various positions decide the size of other atypical substrates (Hove and Bhave, 2011). The effect of other putative regulatory factors like intracellular pH, cations, and inhibitors on the function of AQPs has also been suggested (Maurel et al. 2015). Gating of AQPs has been shown to be regulated by intracellular pH, providing a rapid mechanism to adjust membrane water transport in response to changing environmental conditions (Tournaire-Roux et al. 2003). In several plant aquaporins, pH-dependent gating has been associated with conserved histidine residues and is considered an intracellular pH sensor (Törnroth-Horsefield et al. 2006; Leitao et al. 2012; Frick et al. 2013b). Furthermore, various environmental stresses such as water scarcity, salinity and nutrient imbalance have been shown to alter AQPs’ expression level (reviewed by Kapilan et al. 2018; Sun et al. 2024).

Despite extensive research on AQPs in model plants, studies in cork oak are limited. The release of the full genomic sequence of *Q. suber* (Ramos et al. 2018) revealed the presence of putative AQP-encoding genes available at Cork Oak Genome Portal (http://corkoakdb.org), but their functional significance is still poorly understood. In other *Quercus* species, different AQPs have been shown to contribute to water homeostasis in various tissues and conditions. In *Q. macrocarpa*, two among the four identified putative PIP AQPs showed higher transcript abundance in shaded leaves, suggesting a role in water homeostasis under different light conditions rather than in the rapid hydraulic response (Voicu et al. 2009). In *Q. petraea* and *Q. robur*, nine AQPs belonging to the PIP and TIP subfamilies displayed differential expression across developmental root zones, indicating tissue- and species-specific regulation associated with root function and environmental adaptation in these species (Rasheed-Depardieu et al. 2012). In Japanese oak species, including *Q. acuta*, *Q. glauca*, *Q. serrata*, leaf major vein blockage and aquaporin inhibition by mercury chloride (HgCl_2_) reduced the leaf hydraulic and stomatal conductance depending on leaf venation type, suggesting that AQPs contribute to leaf water transport in a species-specific manner through interaction with leaf venation architecture (Harayama et al. 2019). In contrast, in *Q. suber,* AQP-related studies remain limited and have largely been restricted to transcriptomic analysis rather than their functional characterization (Miguel et al. 2015; Usié et al. 2017; Mendes et al. 2022). This knowledge gap highlights the need for further functional studies to understand how AQPs contribute to water transport and stress adaptation in cork oak.

To address this gap, we characterized cork oak AQPs’ function, from evolutionary relationships to stress responses, by combining phylogenetic analysis, heterologous functional assays, in situ immunolocalization, and drought-responsive expression analysis. Briefly, cork oak AQPs were cloned and expressed in *aqy-null* strains of *Saccharomyces cerevisiae*. Their water and glycerol transport activities, as well as their regulation, were assessed by stopped-flow spectroscopy. Potential substrate specificity was further explored through yeast growth assays with test substrates. Besides the yeast system, the functional roles of these AQPs were also studied *in planta*. Immunolocalization was performed on young cork oak stems, and quantitative expression analysis under drought stress provided insights into the tissue-specific function of AQPs. This study provides the first comprehensive evidence of cork oak AQPs’ functional roles and regulation, offering insights into cork oak adaptation to drought and paving the way for future conservation strategies in the context of climate change.

## Materials and methods

### Plasmid, yeast strain, and culture conditions

Putative cork oak AQPs were expressed using the pUG35 plasmid (Güldener and Hegemann 1998) in the *aqy-null* YSH1172 *S. cerevisiae* strain (Δ*AQY1*/*AQY2*), following our previously established protocol (Sabir et al. 2014, 2020b). Yeast transformants were selected and cultured in amino-acid-free Yeast Nitrogen Base (YNB) (DIFCO) with 2% (w**/** v) glucose supplemented to support prototrophic growth (Pronk 2002).

### Analysis of sequences and expression of cork oak AQPs in yeast

Multiple sequence alignment of AQPs sequences from *Q. suber* with other *Quercus* species and with other model plants like *Arabidopsis thaliana*, *Vitis vinifera*, *Nicotiana tabacum*, *Zea mays*, and *Glycine max* was performed using Clustal X (Thompson et al. 1997) in BioEdit (Hall 1999). The phylogenetic tree using the resulting sequence alignment was generated in MEGA X employing the neighbor-joining method with 1000 bootstrap replicates using with the JTT Model (Kumar et al. 2018).

Conservation of crucial residues in a particular region was represented by logotypes of the conserved regions (Crooks et al. 2004). Selected sequences of equal length were aligned by ClustalW and the consensus profile was represented through a logotype (http://weblogo.threeplusone.com/create.cgi) (Schneider and Stephens 1990; Crooks et al. 2004). Incomplete or longer sequences were not used in some of the logotype depictions.

Cloning and expression of cork oak AQPs were performed by our previously mentioned protocol for grapevine AQPs (Sabir et al. 2014, 2020b). Briefly, full-length cork oak AQPs (*QsPIP2;4*, *QsTIP2;1*, *QsNIP1;2* and *QsNIP6;1*) were amplified from cDNA of *Q. suber* using the designed primers mentioned in Table S1. Cloning and expression of the obtained *AQP* amplicons were done by our previously established methods (Sabir et al. 2014, 2020b). Briefly, the pUG35 plasmid construct with GFP-tagged *QsAQPs* was transformed in *aqy-null S. cerevisiae* strain, and the localization of cork oak AQPs in the yeast membrane was done under a Leitz Wetzlar, Germany 513,558 epifluorescence microscope, and images were captured as mentioned in our previous studies (Sabir et al. 2014, 2020b).

Obtained amino acid sequences were analyzed to confirm the typical characteristics of the MIPs family for membrane topology and hydrophobicity using various ExPASy tools (Hofmann and Stoffel 1993a, 1993b; Tusnady and Simon 2001) and were compared with other plant AQPs using the NCBI BLASTP tool.

### Water and glycerol transport assays

Water and glycerol transport through cork oak AQPs expressed in yeast strains were assessed using stopped-flow fluorescence spectroscopy (HI-TECH Scientific PQ**/** SF-53) following previously established protocols (Soveral et al. 2007; Sabir et al. 2020b). Briefly, yeast strains were preloaded with 5-(and-6)-carboxyfluorescein diacetate (CFDA), which is hydrolyzed by cellular esterases yielding CF, the volume-sensitive fluorophore that stays trapped inside the cells. Cells were then subjected to a hyperosmotic shock with sorbitol inducing water outflux and cell shrinkage with subsequent changes in fluorescence output. The rate constant (k) of cell volume change was obtained by fitting the fluorescence signals to a single-exponential to determine the water permeability coefficient (*P*_*f*_), using the formula *P*_*f*_ = k (V_o_/A)(1/V_w_(osm_out_)_*∞*_), where V_w_ represents the molar volume of water, V_o_/A is the initial ratio of cell volume to area, and (osm_out_)_*∞*_ denotes the final osmolarity of the medium after the osmotic shock. For the evaluation of glycerol permeability, a hyperosmotic shock with glycerol resulted in fast cell shrinkage followed by re-swelling due to glycerol entry and subsequent water influx. The rate of re-swelling was determined from the slope of a linear regression. The glycerol permeability coefficient (*P*_*gly*_) was determined from *P*_*gly*_ = m (V_o_/A), where m is the linear slope obtained from the glycerol influx signal following the previously described protocol (Sabir et al. 2020b).

Activation energies (*E*_*a*_) for water and glycerol transport were estimated using permeability coefficients (*P*_*f*_ or *P*_*gly*_) measured at different temperature ranges (10 °C–35 °C) and determined from the slope of an Arrhenius plot of ln*P*_*f*_ or ln*P*_*gly*_ versus 1**/** T, respectively, as previously described (Soveral et al. 2007; Sabir et al. 2020b).

### Effect of mercury chloride and site-directed mutagenesis

To analyze the effect of mercury on the cloned AQPs’ activities, cells were treated with 0.5 mM HgCl_2_ for 15 min at room temperature prior to the osmotic shock, as explained previously (Sabir et al. 2014, 2020b).

The Cys69 residue is considered to potentially bind Hg^2+^ and where disulfide bridges are likely to be established between different monomers (Bienert et al. 2012). To establish the role of Cys69 in PIPs for binding to mercury, a C69A mutant was constructed by replacing with alanine, an amino acid with a short and very unreactive side chain. Mutated variants of the aquaporin QsPIP2;4A were generated by PCR-based site-directed mutagenesis with specific complementary primers containing the desired point mutations (C69A) (Table S1). Each set of primers was used to amplify the plasmid pUG35 containing the QsPIP2;4A, followed by digestion of the methylated parental plasmid DNA with DpnI enzyme as mentioned before (Sabir et al. 2020a). After confirmation by sequencing, the selected plasmids were transformed into the yeast strain, and the mutated cork oak AQPs were localized in the yeast membrane by following the method described above in the section of analysis of sequences and expression of cork oak AQPs in yeast.

### pH-dependent gating of cork oak AQPs

pH-dependent gating of cloned AQPs was evaluated by performing permeability assays under different external pH (5.0 and 6.8) as described previously (Leitao et al. 2012; Sabir et al. 2020b). Incubation and osmotic shock solutions were adjusted to the desired pH. To examine the role of intracellular pH on AQPs’ gating, cytosolic acidification was induced by adding 4 mM benzoic acid to the pH 5.0 solutions. Relative abundance of labeled ^14^[C]-propionic acid was measured to calculate the intracellular pH (pH_in_) of yeast cells (Pampulha and Loureiro-Dias 1989).

### Substrate-specific growth assays of yeast cells expressing cork oak AQPs

Putative transport of substrates beyond water and glycerol through cork oak AQPs was determined by a yeast growth sensitivity test in the presence of the test substrates like boric acid (20, 40, and 60 mM), sodium arsenite (0.5, 1.0, 1.5 mM) and hydrogen peroxide (0.25, 0.5, 0.75 mM) by following the protocols described before (Sabir et al. 2014, 2020b). Briefly, selected yeast strains were harvested at the initial log-growth stage at an optical density corresponding to OD_640nm_ = 0.6–0.8, washed and resuspended to an OD_640nm_ = 10. Serial dilutions were spotted onto YNB agar plates containing test substrates. Growth differences were recorded after 1–2 weeks at 28 °C.

### Immunodetection of AQPs in cork oak tissues

#### Tissue fixation and sectioning

Stems of one-year-old cork oak plants were collected and processed as described by Inácio et al. (2018). In detail, stems were cut into 1 cm long pieces with a fresh razor blade and immediately fixed in 4% paraformaldehyde prepared in phosphate-buffered saline (PBS, pH 7.4) under vacuum and incubated overnight at 4 °C. Samples were dehydrated through a series of graded ethanol solutions (50–100%). Tissues were gradually cleared with a series of histoclear (VWR Chemicals) and ethanol solutions (50% ethanol/50% histoclear, 25% ethanol/75% histoclear, and 100% histoclear) and finally embedded in paraffin (VWR Chemicals). Tissues embedded in paraffin blocks were sliced into 8 μm sections using a rotary microtome (MEDITE M530, Burgdorf, Germany). Sections were mounted on poly-L-lysine pre-coated slides (1 mg ml^−1^, Sigma-Aldrich, Madrid, Spain).

#### Immunolocalization of AQPs

Immunodetection was performed by commercially available antibodies against subgroups PIP2 and TIP2 AQPs as described by Inácio et al. (2018). NIP members were not included for this study due to unavailability of cork oak validated/predicted NIP-specific antibody and putative lower intrinsic expression. Cork oak stem sections were dewaxed and rehydrated with permeabilization solution (89,5% PBS 1X, 0.5% Triton and 10% DMSO) and wash solution (99.5% PBST 1X, 0.5% Tween 20), followed by incubation with an 8% BSA solution prepared in PBS. The sections were incubated overnight with 75 μL of 1:100 diluted primary antibodies either anti-PIP2 or anti-TIP2 (respectively, AS12 2110 and AS12 2619, Agrisera, Vännäs, Sweden) in a humid chamber. The anti-PIP2 can detect PIP2;1, PIP2;2, PIP2;4, PIP2;6 and PIP2;7 and anti-TIP2 can detect TIP2;2 and TIP2;3 (Inácio et al. 2021). The negative control sections were obtained by substituting the primary antibody with buffer PBST. Thereafter, the sections were incubated with goat polyclonal secondary antibody to rabbit IgG—H&L conjugated to Alexa Fluor® 488 (1:100 dilution, AB150077, Abcam, Cambridge, UK). The slides were mounted with DAPI-containing VECTASHIELD Mounting Medium (Vector Laboratories, Burlingame, California, USA) as described by Inácio et al. (2021, 2018). Images were acquired using an epifluorescence microscope (Carl Zeiss) with appropriate filter sets. Z-stack image series with 0.5-μm intervals were captured on an Axio Imager.Z1 and maximum fluorescence projections were generated as previously described (Inácio et al. 2021).

### Effect of drought stress on AQPs in cork oak plants

#### Plant growth conditions and drought stress treatment

Cork oak plants were grown in 3L pots and were well-irrigated with water and mineral solution (10:1, V:V) (Rhue et al. 1978), under controlled conditions with 25/23 °C temperature at day/night, photoperiod of 16 h light/8 h dark (fluorescent lamps, Gro-Lux F18W/GRO), irradiance of 300 µmol quanta m^−2^ s^−1^ in a walk-in growth-room.

Well-watered (WW) plants were irrigated to full capacity every other day, whereas water deficit stress (WS) was imposed by withholding irrigation (day 0) for ten days followed by rewatering (R1) to full capacity, similar to well-watered (WW) conditions. Leaves, stems and roots were collected at different time points for further analysis. The upper 4–5 leaves and their attached stems were designated as young leaves and young stems, respectively, while those below this section were classified as mature tissues.

Leaf water potential (Ψ_w_) was measured using a pressure chamber (Model 600, PMS Instruments Company, Albany, OR). At day 0, day 10, and recovery, the third fully expanded leaf of each plant was selected to measure the photosynthetic rate at ambient CO_2_ (A), stomatal conductance (gs), and transpiration rate (E) using a CIRAS-3 Portable Photosynthesis System (PP-Systems, Amesbury, MA, USA) connected to a PLC4 Universal Leaf Cuvette with an 18 mm diameter interchangeable head plate. Intrinsic water use efficiency (WUE) was calculated by dividing A by gs.

#### Differential expression analysis of cork oak AQPs

Total RNA from cork oak tissues was extracted using the Spectrum Plant Total RNA kit (Sigma–Aldrich, St. Louis, USA), following the manufacturer’s instructions. RNA concentration was quantified through spectrophotometry in a Synergy HT Multiplate Reader (BioTek, Friedrichshall, Germany) with a Take3 Multi-Volume Plate (BioTek), with Gen5 software (BioTek). Sample purity was evaluated based on the A260/A280 ratio, accepting only samples within the 1.8–2.1 range. Further, the integrity of RNA was verified by 2% agarose gel electrophoresis, confirming the presence of two clear ribosomal RNA bands.

cDNA was synthesized using 0.5 µg of total RNA, oligo-dT primers (STABvida, Caparica, Portugal) and RevertAid Reverse Transcriptase kit (Thermo Fisher Scientific, Waltham, MA, USA), according to the manufacturer’s instructions. The synthesized cDNA was stored at –20 °C until further use.

Relative expression of selected *AQPs* (*PIP2;4*, *TIP2;1, NIP1;2* and *NIP6;1*) was quantified by real-time quantitative PCR (RT-qPCR) in a CFX Connect Real Time PCR (Bio-Rad, Hercules, CA, USA) using 96-well transparent reaction plates. Specific primers were designed with the Integrated DNA Technologies OligoAnalyzer tool (https://eu.idtdna.com/calc/analyzer), and their sequences are mentioned in Table S1. PCR reactions were performed in 20 µl volumes containing EvaGreen Master mix (SsoFast_EvaGreen supermix, Bio-Rad), primers (10 µM), diluted cDNA (2.5 ng µl⁻^1^), and nuclease-free Mili-Q water. The following cycling conditions were optimized for amplification: initial denaturation at 95 °C for 3 min, 40 cycles of denaturation (95 °C), annealing (60 °C for 30 s), and extension (72 °C for 45 s), with fluorescence acquisition at the end of each cycle. A final extension step at 72 °C for 5 min was included to complete the amplification.

Logarithmic amplification plot of the normalized fluorescence signal (ΔRn) was generated by subtracting the baseline obtained between cycles 5 and 17. The fluorescence threshold (Rn) of 68 was applied to determine the quantification cycle (Cq) values, which were then exported to Excel for subsequent quantification. Relative transcript abundance was calculated using the ΔΔCq method, normalizing target gene expression against the reference gene *GAPDH*. Three biological replicates and two technical replicates per sample were analyzed to ensure reproducibility and statistical reliability.

### Statistical analysis

All measurements were obtained from a minimum of three independent biological replicates. For stopped-flow experiments, each selected condition typically involved five hyperosmotic shocks per temperature, with at least ten shocks conducted at 23 °C for each experiment. The resulting stopped-flow spectroscopy data were evaluated using Welch’s t-test, which accounts for unequal variances. Statistically significant differences are marked by asterisks: * for *p* < 0.05, ** for *p* < 0.01, and *** for *p* < 0.001. Results are shown as mean ± standard deviation (SD) or standard error of the mean (SE), as specified where appropriate.

## Results

### Cloning and expression of Quercus AQPs

**Fig.1:**
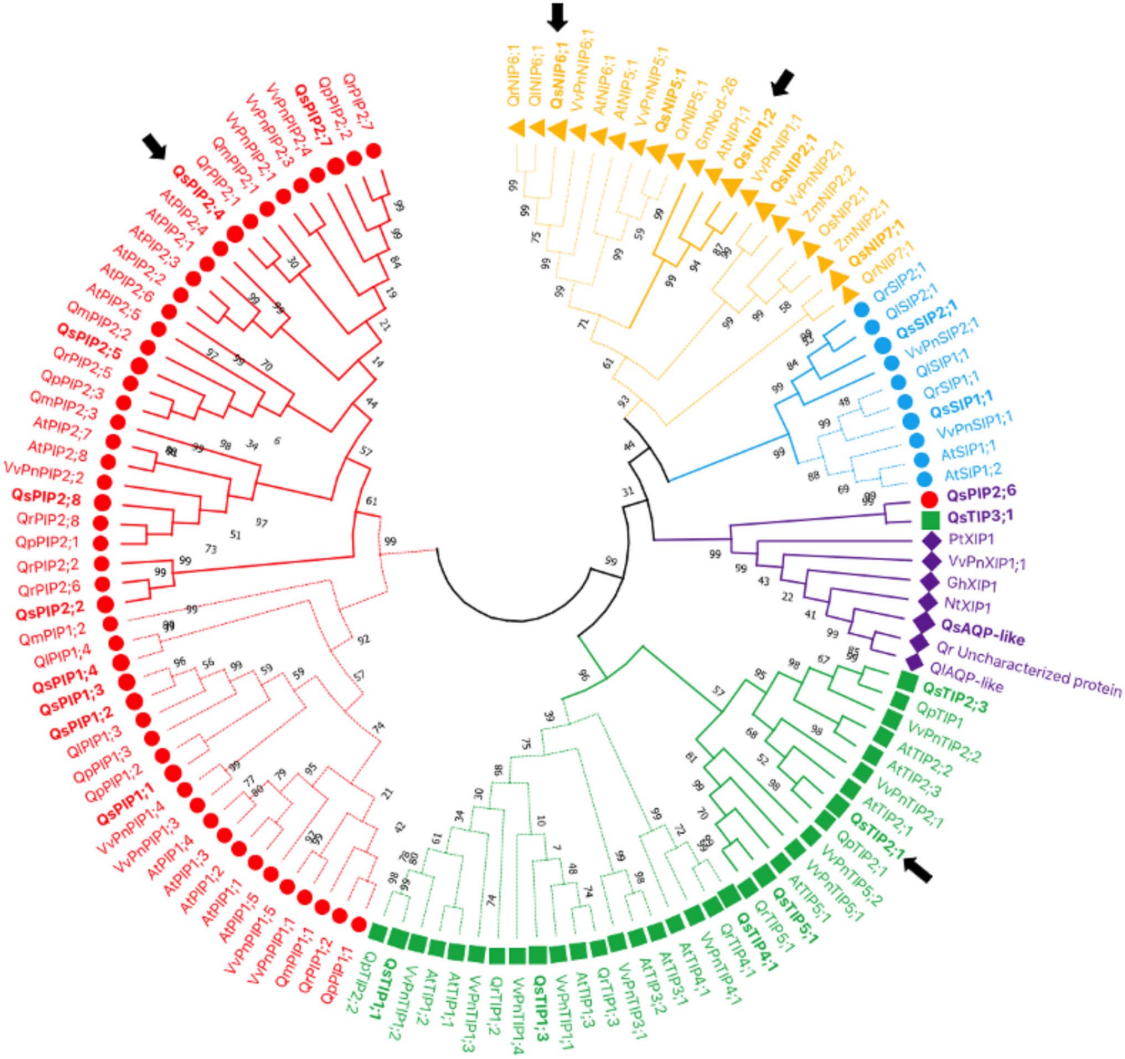
Phylogeny of cork oak AQPs. The phylogenetic relationship between cork oak AQPs (bold) and other *Quercus* species and reference plant AQPs was analyzed by aligning the total of 122 AQP amino acid sequences. The AQPs selected in this study to clone and characterize are marked with an arrow. The tree was constructed by MEGA X software using the neighbor-joining method with 1000 bootstrap replicates (Kumar et al. 2018). Different colors indicate the distinct AQP groups: PIPs are shown as red circles, TIPs as green squares, NIPs as orange triangles, SIPs as blue circles and XIPs as purple diamonds. Subgroups in each family are represented by solid or dashed lines. To identify the origin of each AQP, a species abbreviation is written at the beginning of the protein name as prefix; *At* *Arabidopsis thaliana*, *Gm* *Glycine max*, *Nt Nicotiana tabacum,* *Os* *Oryza sativa*, Pt *Populus trichocarpa*, *QI Quercus lobate*, Qm *Quercus macrocarpa*, Qp: *Quercus patraea*; Qr *Quercus robur*, *Qs Quercus suber*, *Vv* *Vitis vinifera*, *Zm* *Zea mays*

The phylogenetic relationship between cork oak AQPs and those of other *Quercus* species and of reference plant AQPs was analyzed by aligning a total of 122 amino acid sequences. The obtained phylogenetic tree showed a clear distribution of cork oak AQPs into five subfamilies: PIPs, TIPs, NIPs, SIPs, and XIPs (Fig. 1).

The PIPs are the largest subfamily, consisting of ten members, which are grouped into PIP1 and PIP2 subgroups alongside *Arabidopsis* and grapevine PIPs. The TIPs are the second largest subfamily, with seven members, categorized into TIP1 and TIP2 subgroups. The NIPs subfamily comprises five members, further classified into NIP-I, NIP-II, and NIP-III subgroups based on reference plant sequences. Only two members of cork oak SIPs were identified, and these were clustered in the SIP1 and SIP2 subgroups. In the XIP subfamily, three cork oak AQPs were clustered, one of which is labeled as aquaporin-like, while the other two are currently annotated as PIP2;6 and TIP3;1. However, our phylogenetic analysis shows that these sequences cluster with the XIP subfamily. Therefore, more comprehensive phylogenetic and structural analyses are required for definitive subfamily classification for these specific isoforms.

**Fig. 2:**
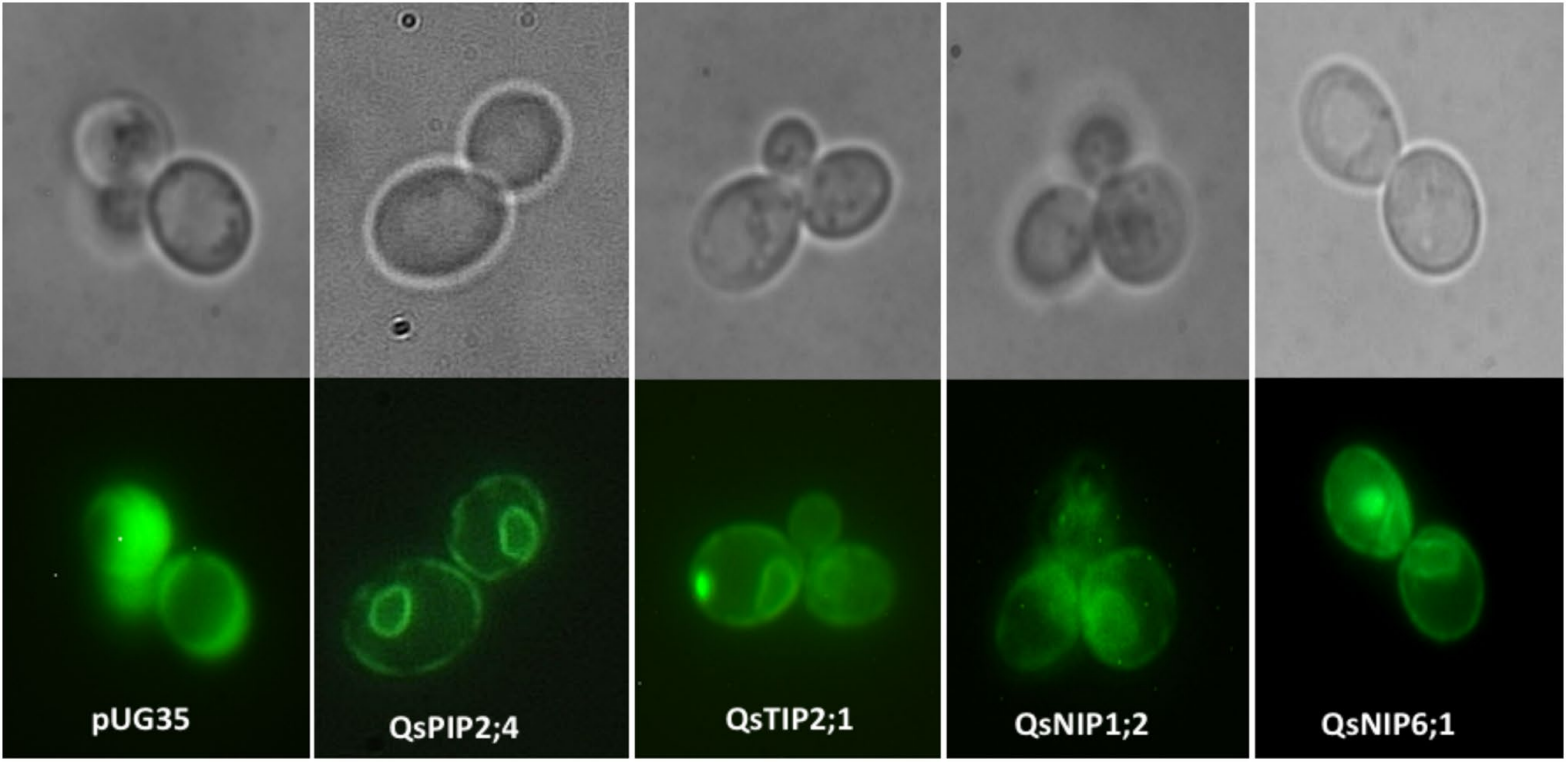
Localization of GFP-tagged cork oak AQPs in the membranes of *aqy-null S. cerevisiae* cells. The control yeast strain was transformed with the empty pUG35 vector, and the expressing strains were transformed with plasmids containing the cork oak AQPs genes. Cells were analyzed under phase contrast microscopy (top panel) and fluorescence microscopy (bottom panel) at 100 × magnification

In the present study, four cork oak AQPs, including QsPIP2;4, QsTIP2;1, QsNIP1;2, and QsNIP6;1, were selected based on their placement in distinct aquaporin subfamilies. These AQPs represent different predicted functional classes, allowing us to examine whether phylogenetic grouping is reflected in their functional role. Full-length cDNAs encoding the selected cork oak AQPs were obtained, and GFP-tagged cloned AQPs were localized in the plasma membrane of the yeast cells. In the control strain (containing empty plasmid pUG35), GFP signals were observed in the cytosol (Fig. 2).

The topology of the deduced protein sequences exhibited conserved characteristics of the AQPs family consisting of six transmembrane helices and five connecting extra- and intracellular loops. The sequences were analyzed using the BLASTP tool, which showed 100% identity with database sequences of cork oak, except for QsPIP2;4, which exhibited 95% similarity (NCBI accession number: POF11915). Sequencing results confirmed that the cloned QsPIP2;4 has a 39 bp nucleotide insertion between positions 520 and 558 (Fig. S1A). The sequence data of cloned QsPIP2;4 in this study has been submitted to the GeneBank database under accession number PV007186. The nucleotide insertion resulted in a longer protein sequence (281 aa) compared to the database sequence length (268 aa). In fact, protein sequence alignment with other PIPs clearly revealed a thirteen-amino acid gap in the database sequence, confirming the omission during the original annotation process (Fig. S1B). Additionally, two selected QsPIP2;4 transformants (clone A and clone B) exhibited an amino acid substitution, with clone B showing a Thr70Ser change (Fig. S2).

All amino acid sequences obtained from *Q. suber* demonstrated significant similarities with those of other *Quercus* species, including *Q. lobata*, *Q. macrocarpa*, *Q. patraea*, and *Q. robur*, with identities ranging from 94 to 98%. In comparison, the analysis of AQPs from other plant species revealed high protein identity with *Castanea mollissima*, another member of the Fagaceae family, with identities between 92 and 98% (Table S2). Additionally, these sequences exhibited notable similarity to those of other long-lived woody trees, such as *Corylus avellana* (92%) and *Juglans regia* (96%).

### Functional characterization of cork oak AQPs

#### Water and glycerol transport activities

**Fig. 3:**
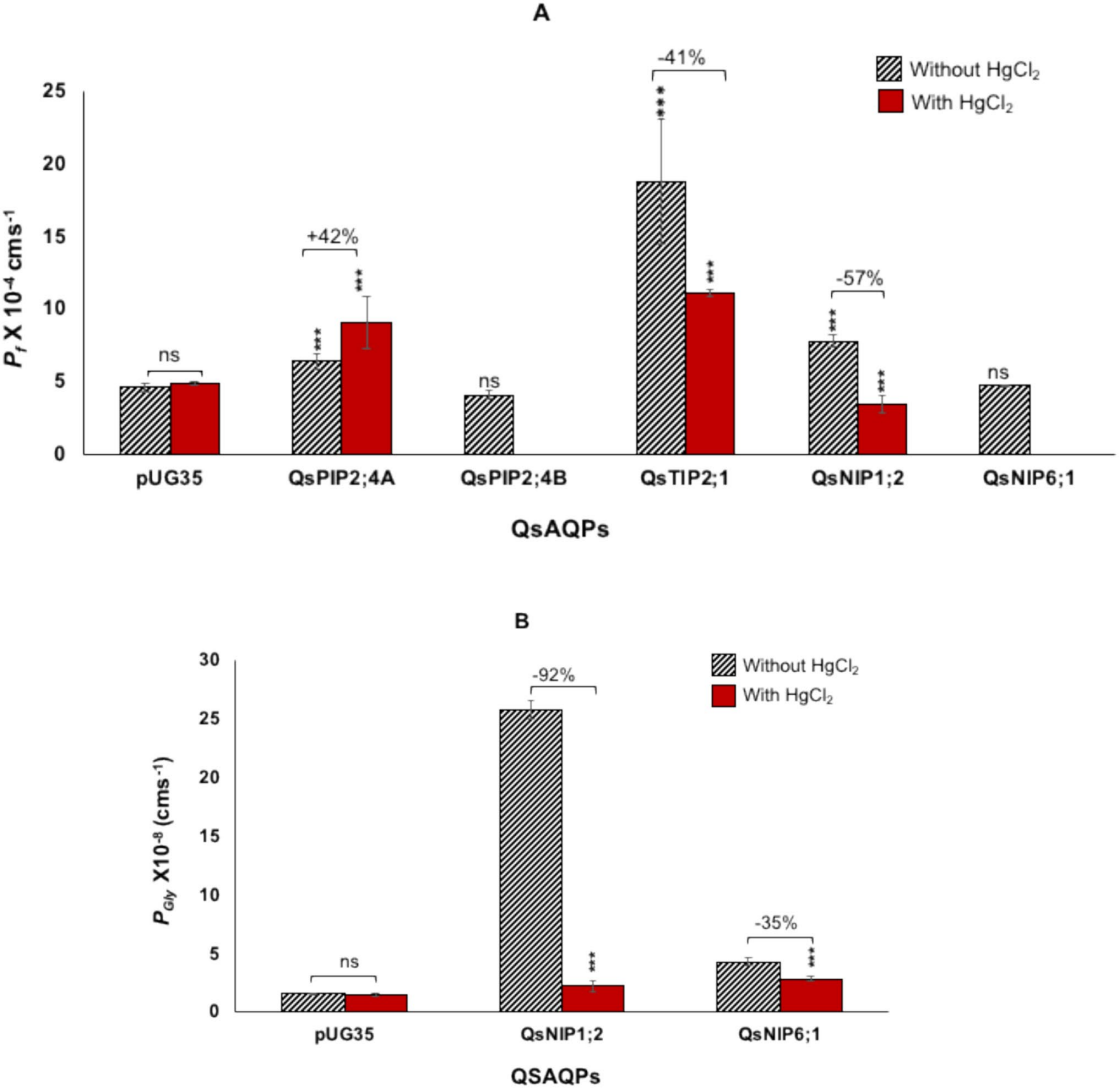
Water (*P*_*f*_) and glycerol (*P*_*gly*_) permeability coefficients of cork oak AQPs measured by stopped-flow spectroscopy. Effect of mercury chloride on (**A**) Water and (**B**) Glycerol permeabilities in yeast expressing cork oak AQPs (red bars). Data are presented as the mean ± SD from a minimum of three independent experiments, each consisting of ten traces. Statistically significant differences are indicated with asterisks, determined using the Welch’s t-test (* *p* < 0.05, ** *p* < 0.01, and *** *p* < 0.001)

The water and glycerol transport activities of cork oak AQPs were analyzed using stopped-flow spectroscopy. The water permeability coefficient (*P*_*f*_*)* was determined in all the yeast strains expressing cork oak AQPs, whereas activation energies (*E*_*a*_) for water transport were estimated in strains that exhibited higher water permeabilities than the control strain expressing empty plasmid pUG35. Expression of QsPIP2;4, QsTIP2;1 and QsNIP1;2 AQPs significantly increased the water transport activity of the yeast strains (Fig. 3A, Table 1). Among the cloned AQPs, QsTIP2;1 demonstrated the highest water permeability coefficient (18.7 ± 4.3 × 10^−4^ cm s^−1^) compared to the control strain (4.6 ± 0.3 × 10^−4^ cm s^−1^) (Fig. 3A) along with a decrease in activation energy from 14.05 kcal mol^−1^ to 7.3 kcal mol^−1^ (Table 1). For QsPIP2;4, two transformants [clone A and clone B (with Thr70Ser)] were selected to measure the water permeability. Expression of clone B2 could not increase the *P*_*f*_ (4.1 ± 0.27 × 10^−4^ cm s^−1^), whereas clone A2 showed higher water permeability (6.36 ± 0.5 × 10^−4^ cm s^−1^) and lower activation energy (11.47 kcal mol^−1^). Among the two cloned NIPs, only QsNIP1;2 exhibited higher *P*_*f*_ (7.8 ± 0.43 × 10^−4^ cm s^−1^) and lower *Ea* (12.3 kcal mol^−1^), while QsNIP6;1 did not improve water permeability (4.67 ± 0.05 × 10^−4^ cm s^−1^) in the yeast strain (Fig. 3A, Table 1).

**Table 1:** Activation energy (*E*_*a*_) for water and glycerol transport through functional cork oak AQPs at different intracellular pH

Strains	Activation energy for water transport *E*_*a*_ (kcal mole^−1^)			Activation energy for glycerol transport *E*_*a*_ (kcal mole^−1^)		
	pH 5.0 (pH_in_ 6.1)	pH 5.0 + BA (pH_in_ 4.8)	pH 6.8 (pH_in_ 6.8)	pH 5.0 (pH_in_ 6.1)	pH 5.0 + BA (pH_in_ 4.8)	pH 6.8 (pH_in_ 6.8)
pUG35	14.05	13.8	13.67	24.3	25.1	24.2
QsPIP2;4	11.47	13.2	10.1	nd	nd	nd
QsTIP2;1	7.3	7.29	7.16	nd	nd	nd
QsNIP1;2	12.3	13.6	9.8	6.87	16.52	9.72
QsNIP6;1	nd	nd	nd	10.1	9.24	9.85

^*^nd-not determined

Expression of both cloned NIPs (QsNIP1;2 and QsNIP6;1) significantly enhanced glycerol transport in the yeast strains. Both QsNIP1;2 and QsNIP6;1 showed higher glycerol permeability coefficient (*P*_*gly*_) and lower activation energies for glycerol (Fig. 3B, Table 1). A markedly higher glycerol permeability (25.84 ± 0.77 × 10^−8^ cm s^−1^) with concomitant reduced activation energy (6.87 kcal mol^−1^) was measured, representing a 16.7-fold increase in glycerol flux compared to strains transformed with the empty vector (*P*_*gly*_: 1.55 ± 0.11 × 10^−8^ cm s^−1^, *Ea*: 24.3 kcal mol^−1^) (Fig. 3B, Table 1). In contrast, QsNIP6;1 expression resulted in a 183% increase in glycerol permeability (4.38 ± 0.38 × 10^−8^ cm s^−1^) along with lower activation energy (10.1 kcal mole^−1^) (Fig. 3B, Table 1).

#### Effect of mercury chloride on AQPs activity

Mercury chloride (HgCl_2_) is known to inhibit AQPs (Maurel et al. 2015). To assess its impact on both water and glycerol transport, a 0.5 mM HgCl₂ solution was applied to the yeast cells expressing cork oak AQPs, and the channel activity was evaluated using stopped-flow spectroscopy following a 15-min incubation at room temperature as described previously (Sabir et al. 2020b).

As expected, water permeability was reduced by 41% and 57% in yeast strains expressing QsTIP2;1 and QsNIP1;2, respectively (Fig. 3A). Similarly, glycerol permeabilities in the QsNIP-expressing strains were also decreased in response to HgCl₂ treatment (Fig. 3B). Notably, QsNIP1;2 showed significantly reduced water permeability (2.23 ± 0.45 × 10⁻⁸ cm s⁻^1^), indicating a 92% decrease, and QsNIP6;1 exhibited a 35% reduction in glycerol permeability, measured at 2.28 ± 0.22 × 10⁻⁸ cm s⁻^1^ (Fig. 3B).

**Fig. 4:**
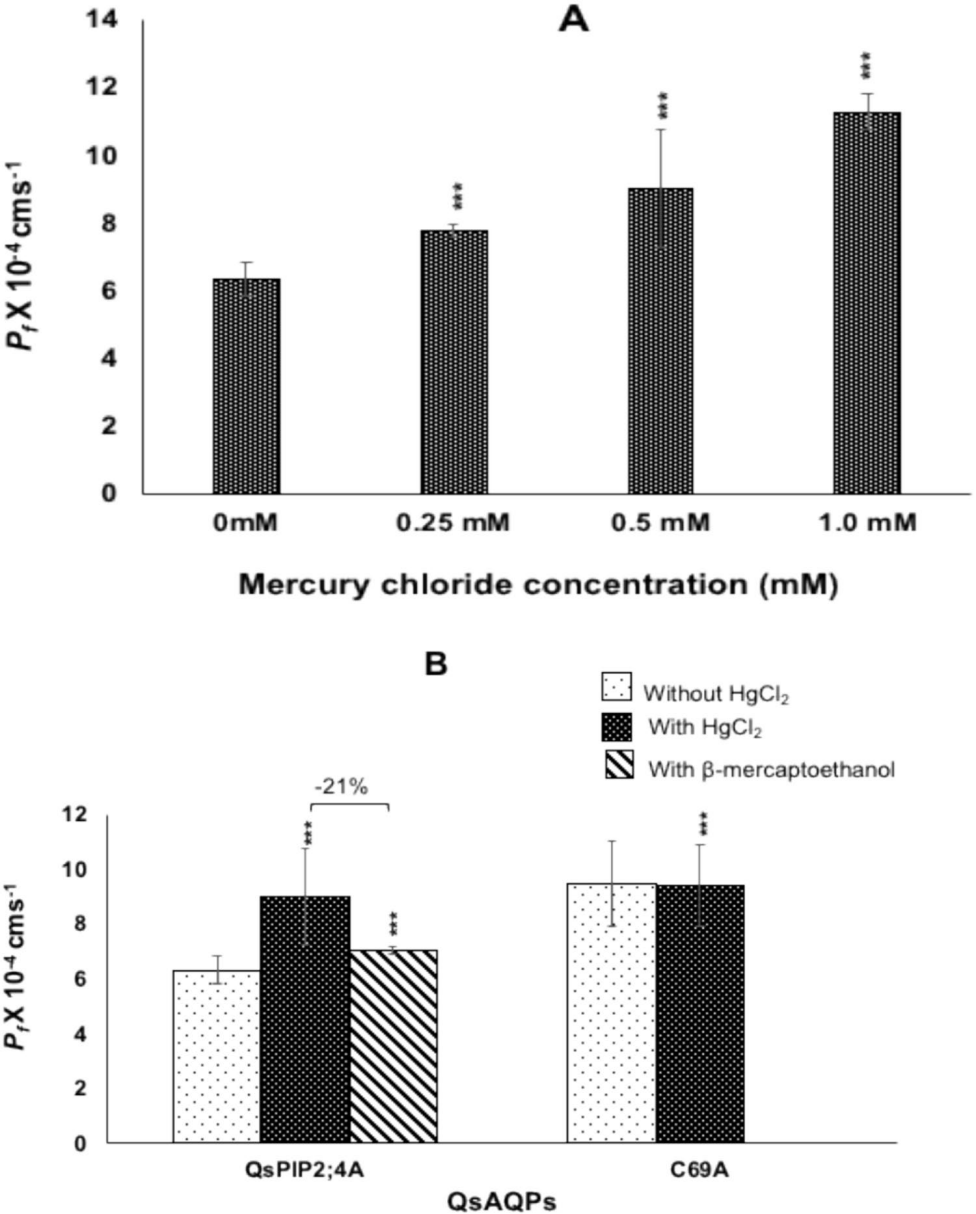
Activation of QsPIP2;4A by mercury chloride. **A** A dose-dependent increase in permeability of PIP2;4A **B** Comparison of water permeabilities between untreated and mercury chloride-treated cells expressing wild-type QsPIP2;4 or the C69A mutant. The activation of QsPIP2;4A was reversed by β-mercaptoethanol. Results are expressed as the mean ± SD from a minimum of three independent experiments, each consisting of ten traces. Statistically significant differences are indicated with asterisks, determined using Welch’s *t*-test (* *p* < 0.05, ** *p* < 0.01, and *** *p* < 0.001)

In contrast, water permeability through QsPIP2;4A unexpectedly increased in the presence of mercury chloride (Fig. 3A). The *P*_*f*_ value of QsPIP2;4A increased from 6.34 ± 0.5 × 10⁻^4^ cm s⁻^1^ to 9.03 ± 1.76 × 10⁻^4^ cm s⁻^1^, resulting in a 42% increase in total water transport. To determine if the increase in water permeability was dose-dependent, the cells were incubated in varying concentrations of mercury chloride (0.25 mM, 0.5 mM, and 1.0 mM) for 15 min prior to the hyperosmotic shock (Fig. 4A). The water permeability increased gradually, from 7.8 ± 0.19 × 10⁻^4^ cm s⁻^1^ (at 0.25 mM HgCl₂) to 11.3 ± 0.51 × 10⁻^4^ cm s⁻^1^ (at 1.0 mM HgCl₂), confirming a dose-dependent response (Fig. 4A).

A conserved cysteine residue in loop A (Cys69) has been suggested as the mercury-binding site and for its role in conformational stability (Savage and Stroud 2007; Törnroth-Horsefield et al. 2006). To investigate this, a C69A mutant of QsPIP2;4 was constructed. Interestingly, the C69A mutant showed a higher water permeability (9.48 ± 1.6 × 10⁻^4^ cm s⁻^1^) than the wild-type QsPIP2;4 (Fig. 4B). However, the water permeability of the C69A mutant remained unchanged upon exposure to 0.5 mM mercury chloride (Fig. 4B). In contrast, when β-mercaptoethanol was added to the cells expressing wild-type QsPIP2;4A, the increase in water transport was reversed, with water permeability decreasing by 21% (7.1 ± 0.14 × 10⁻^4^ cm s⁻^1^) (Fig. 4B).

#### Gating of cork oak AQPs by intracellular pH

**Fig. 5:**
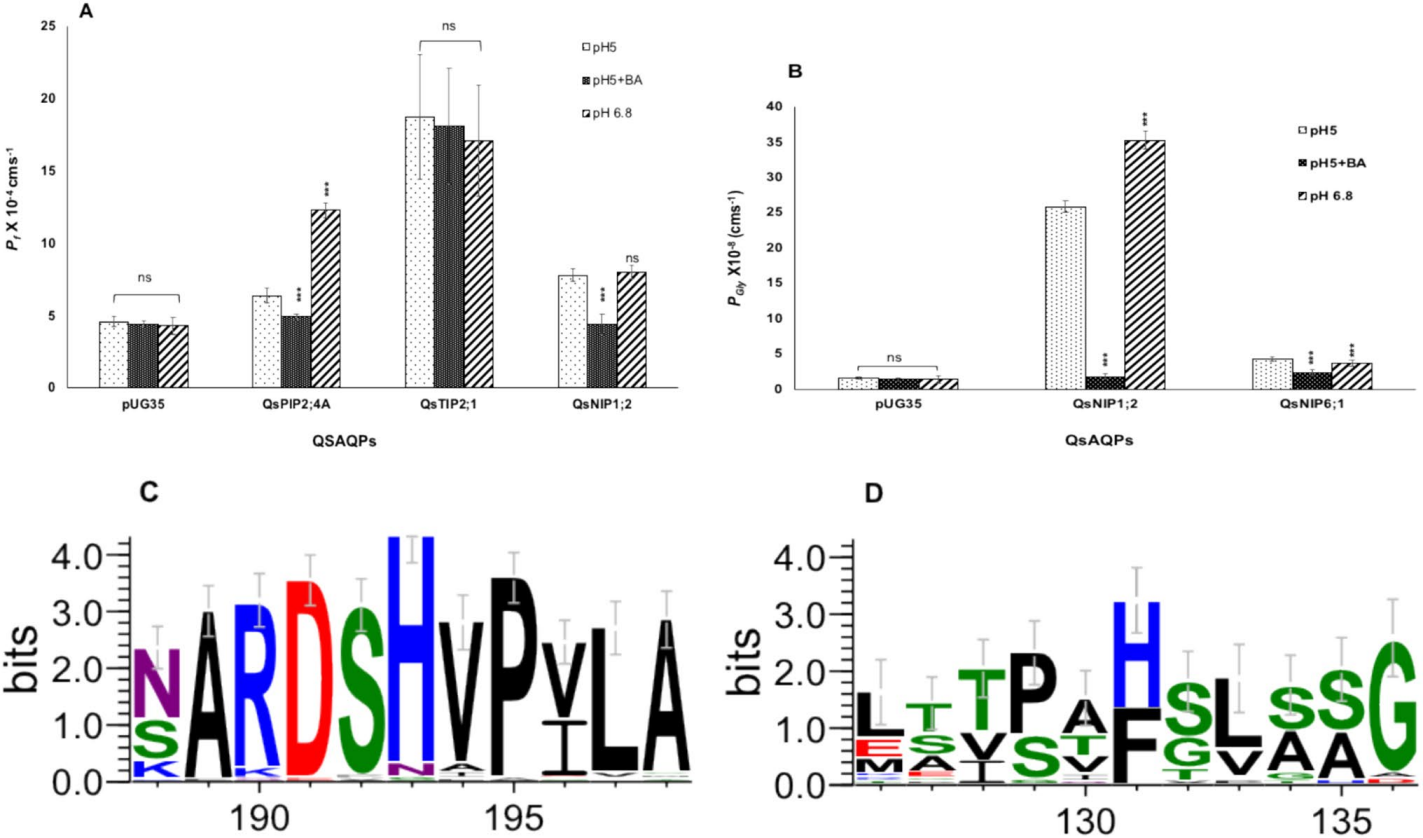
pH-dependent water and glycerol permeabilities, and conservation of putative pH-sensor histidine in cork oak AQPs. **A** Water and **B** glycerol permeability coefficients under different pH conditions, showing pH-dependent regulation of AQPs’ activity. **C** WebLogos of PIPs were derived from the alignment of 51 sequences and **D** TIPs were generated by aligning 24 AQP sequences to depict the relative frequency of residues in loop D. The height of the residues corresponds to their level of conservation. Results are represented as mean ± SD of at least five traces individually obtained from three independent experiments. Statistically significant differences are marked as an asterisk, calculated by Welch’s *t*-test (* *p* < 0.05, ** *p* < 0.01, and *** *p* < 0.001)

The gating of cork oak AQPs by intracellular pH (pH_in_) was evaluated by measuring their water permeability coefficients (*P*_*f*_) and activation energies (*E*_*a*_) at different intracellular and extracellular pH conditions (pH_in_ and pH_out_). When pH_out_ was maintained at 6.8, pH_in_ remained unchanged. However, when pH_out_ was lowered to 5.0, the pH_in_ decreased to 6.1. Further cytosol acidification was achieved by adding 4 mM benzoic acid at pH_out_ 5.0, reducing pH_in_ to 4.8 without affecting pH_out_. The reduction of pH_in_ from 6.8 to 6.1 resulted in a significant decline in both water and glycerol permeability (Fig. 5). Specifically, QsPIP2;4 exhibited a decrease in water permeability from 12.3 ± 0.52 × 10⁻^4^ cm s⁻^1^ to 6.36 ± 0.51 × 10⁻^4^ cm s⁻^1^**,** while QsNIP6;1 displayed a reduction in glycerol permeability from 35.2 ± 1.3 × 10⁻⁸ cm s⁻^1^ to 25.8 ± 0.78 × 10⁻⁸ cm s⁻^1^ (Fig. 5A, B).

The other AQPs showed no significant response to the pH_in_ change under these conditions. Upon further internal acidification with benzoic acid, a marked reduction in water permeability was observed for QsPIP2;4A (4.9 ± 0.2 × 10⁻^4^ cm s⁻^1^) and QsNIP1;2 (4.4 ± 0.73 × 10⁻^4^ cm s⁻^1^) (Fig. 5A). Similarly, glycerol permeability in QsNIP6;1 decreased to 2.3 ± 0.45 × 10⁻⁸ cm s⁻^1^ under acidic conditions (Fig. 5B). Notably, QsNIP1;2 exhibited a more pronounced response, as the glycerol permeability nearly diminished to 1.8 ± 0.34 × 10⁻⁸ cm s⁻^1^ with further internal acidification (Fig. 5B). Conversely, the permeability of QsTIP2;1 remained unaffected across all tested pH conditions (Fig. 5A). The changes in activation energy (*E*_*a*_) for both water and glycerol transport were consistent with the observed variations in permeability, as detailed in Table 1.

Sequence analysis of PIPs and TIPs revealed a conserved histidine (His) residue in loop D, a key determinant of intracellular pH sensitivity. The WebLogo visualization demonstrated the relative frequency of amino acids present in loop D across the analyzed sequences (Fig. 5C and 5D). In PIPs, the His-193 residue is highly conserved, with only a small number of sequences showing asparagine (Asn) substitution (Fig. 5C). In contrast, TIPs exhibit more variability, with some sequences containing phenylalanine (Phe); however, the majority of TIPs, particularly TIP2 isoforms, maintain a conserved His-131 residue (Fig. 5D).

##### Atypical substrate transport

**Fig. 6:**
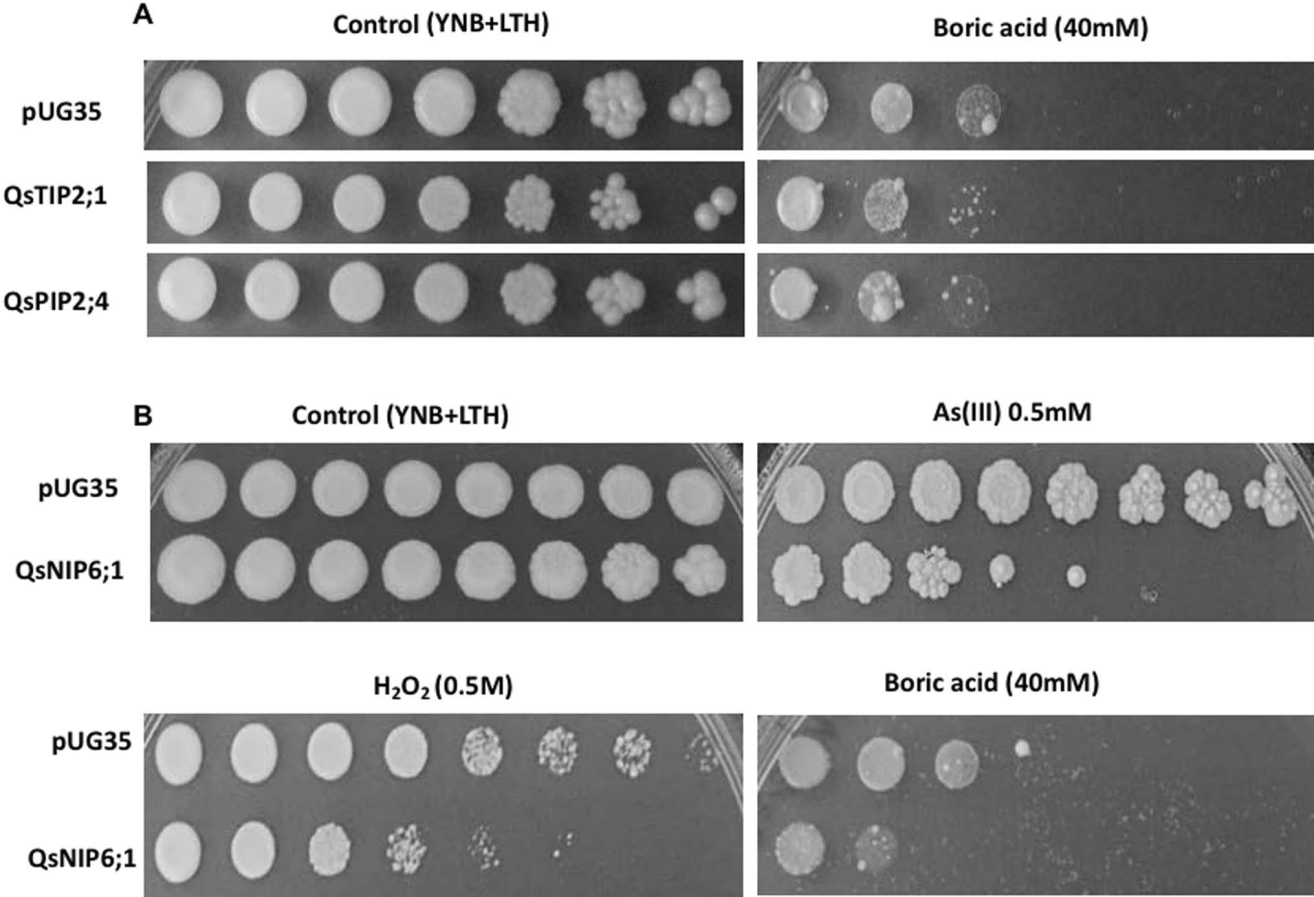
Sensitivity test of yeast strains expressing cork oak AQPs to atypical substrates. Growth patterns of *S. cerevisiae aqy-null* strain either with the empty vector pUG35 or with constructs carrying cork oak AQPs (**A**) QsTIP2;1 and QsPIP2;4, and (**B**) QsNIP6;1 in the presence of boric acid, arsenite, and hydrogen peroxide. The control plate has minimal media without additional substrate. Differences in growth relative to pUG35 cells indicate substrate permeability and sensitivity associated with specific AQPs. Images are representative of a minimum of two separate experiments, each including two replicate plates that produced consistent results

The drop-test growth assay conducted on strains with the cloned cork oak AQPs demonstrated that PIPs and TIPs exhibited a slight sensitivity to boron, supplied as boric acid (40 mM), though this effect was minor compared to the control strain (Fig. 6A). However, these AQPs did not show apparent sensitivity to the other externally supplied substrates, arsenite and hydrogen peroxide (data not shown). In contrast, QsNIP6;1 displayed marked sensitivity, as evidenced by inhibited growth in the presence of these compounds compared to the control strain (Fig. 6B). This phenotype suggests that QsNIP6;1 may have a broader substrate specificity, potentially functioning in the transport or detoxification of toxic substances, such as arsenite and reactive oxygen species, in addition to its role in boron transport.

##### Immunolocalization of PIP2s and TIP2s in cork oak tissues

**Fig. 7:**
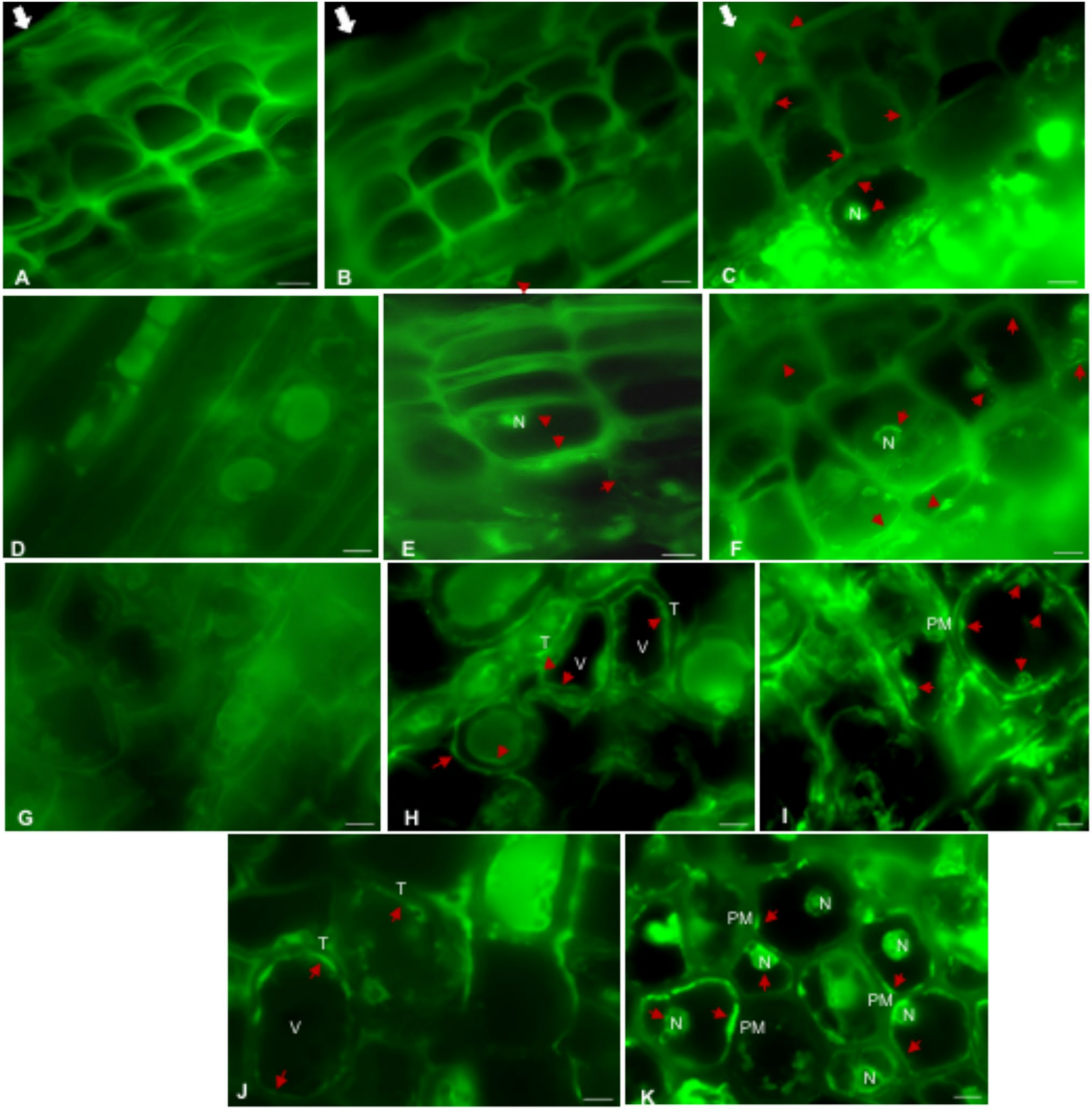
Immunolocalization of TIP2 and PIP2 AQPs in longitudinal sections of cork oak stems. Red arrows highlight the accumulation of AQP signals. **A–C** Outer cork cell layers: white arrows point to the outermost tissue layers: **A** Negative control and **B** TIP2 immunolocalization showed no detectable signals in the outer cork layers, **C** PIP2 immunolocalization revealed faint sporadic signals. **D–F** Inner cork cells and phellogen layer: **D** Negative control sections lacked detectable signal. **E** TIP2 and **F** PIP2 signals increased in the inner cork cells and phellogen layer. **G–K** Cortex/parenchyma layers: **G** Negative control without detectable signals in cortex cells, **H** TIP2 and **I** PIP2 immunolocalization showed their localization on the membranes of cortex cells. Small vesicles are clearly seen protruding into the vacuole. **J** TIP2 was localized on both the vacuolar and plasma membranes. **K** PIP2 signals were observed on the plasma membrane and around the nucleus, likely corresponding to the endoplasmic reticulum. *N* Nucleus, *V* Vacuole, *T* Tonoplast, *PM* Plasma membrane. Bar = 10 μm

To gain insight into the intracellular localization of AQPs in young cork oak plants, we next examined the tissue-specific distribution of the most abundant AQPs (PIPs and TIPs) *in planta* after characterizing their intrinsic transport properties in yeast. The longitudinal section of the one-year-old stem showed six–seven layers of cork cells (Fig. 7A–C). The negative control sections, where only the secondary antibody was applied, displayed general tissue autofluorescence without any detectable AQPs’ signals (Fig. 7A, D and G).

In cork layers, TIP2 and PIP2 were accumulated only in the younger cork cell layers, while in the older and more differentiated cork cell layers lack of signals were observed, eventually complete loss of the signal in the outmost layer of the cork (Fig. 7B and C). The phellogen situated below the cork cells, showed significant accumulation of PIP2 and TIP2 signals, suggesting the presence of these AQPs in this region (Fig. 7E and F). Thereafter signal accumulation was also observed in the phelloderm or cortex layers, which were composed of characteristic round parenchyma cells (Fig. 7G–I). In these cells, intracellular localization of both PIP2 and TIP2 was detected (Fig. 7J and K). TIP2 was localized on the vacuolar membrane (Fig. 7J), whereas PIP2 was prominently localized in the plasma membrane, as well as around the nucleus, likely corresponding to the endoplasmic reticulum (ER) (Fig. 7K). The PIP2 signals were notably more intense compared to those of TIP2.

##### Drought stress in cork oak plants

**Fig. 8:**
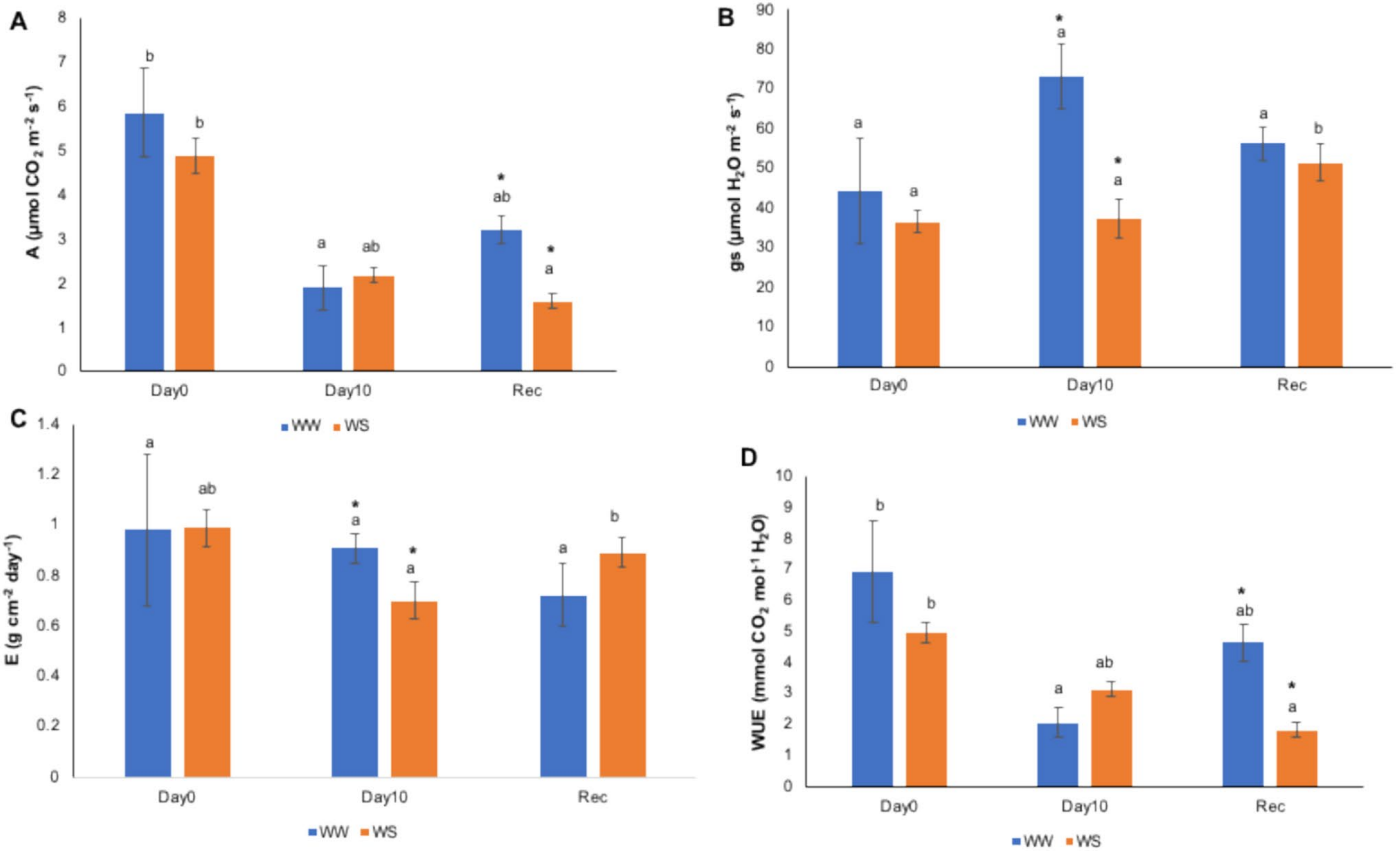
Gas-exchange parameters measured in well-watered (WW) and water stress (WS) cork oak plants during the 10-day water stress assay. Plants were rewatered following 10 days of water stress, and recovery parameters were assessed 72 h after rewatering. Measurements were performed with a CIRAS-3 (PP systems). **A** Photosynthesis rate (A); **B** Stomatal conductance (gs); **C** Transpiration rate (E); **D** Water Use Efficiency (WUE). All values are the mean of three independent samples (*n* = 3). Asterisks represent significant differences between WW and WS for the parameter/time of measurement, and different letters indicate significant differences between time of measurement for the treatment/parameter. In both significant differences, *p* values are < 0.05

We next analyzed the physiological response of cork oak and AQPs’ expression under drought stress to connect the functional characterization in yeast with *in planta* response. Under drought stress, photosynthesis rates decreased in both WW and WS plants after 10 days of monitoring and did not recover in WS plants after rewatering (Fig. 8A). Stomatal conductance (*gs*) and transpiration (E) during stress were higher in WW plants than in WS plants (Fig. 8B, C). Interestingly, WUE was slightly higher in WS plants during stress and in WW plants after rewatering (Fig. 8D).

**Fig. 9:**
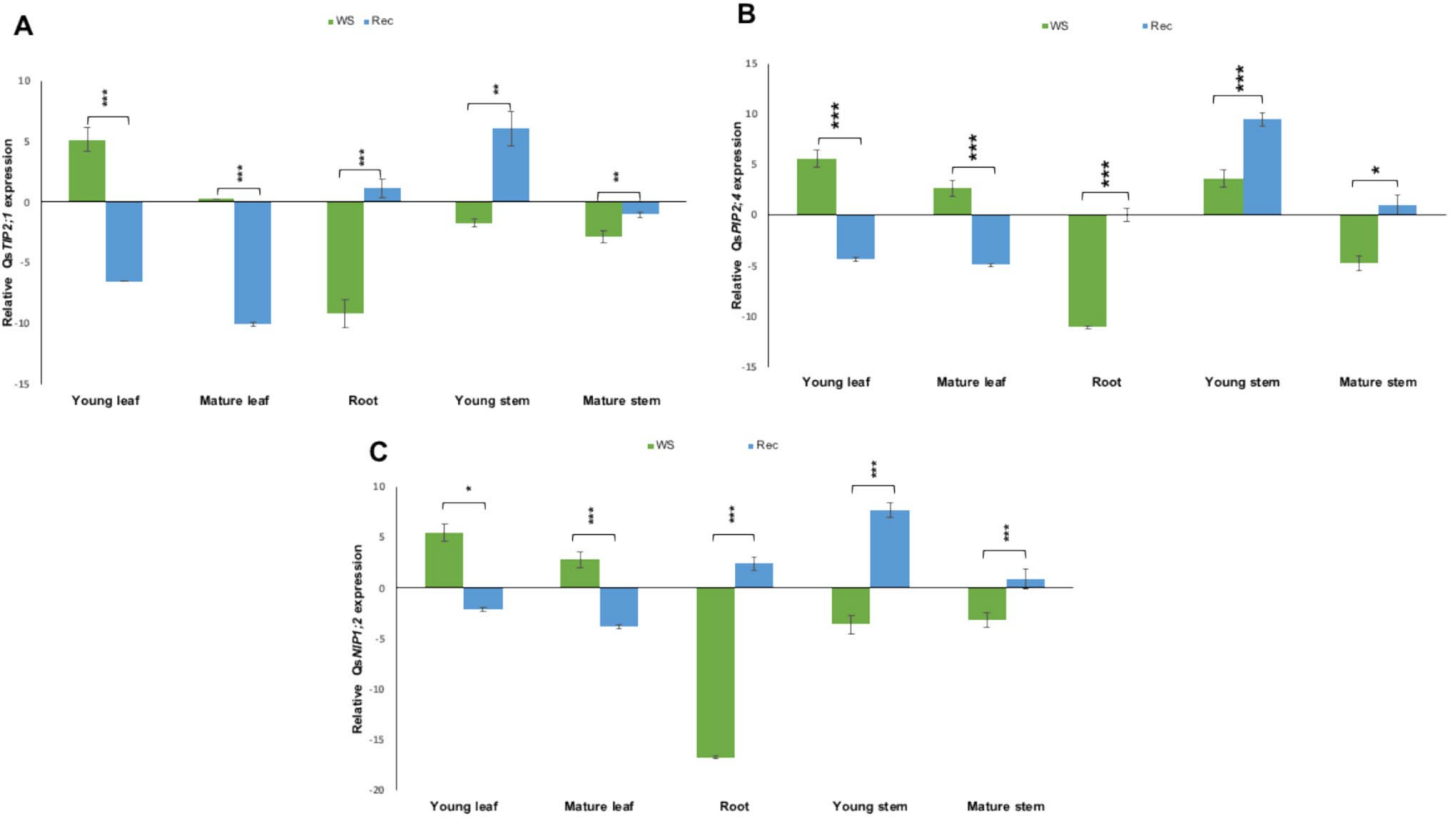
Relative expression of cork oak *AQPs* in young and mature leaves, young and mature stems and roots under drought stress. **A** *QsTIP2;1*, **B** *QsPIP2;4*, **C** *QsNIP1;2*. Expression of *AQPs* in the tissues collected from plants after 10 days of water-stressed (WS) and upon recovery, 72 h after rewatering (Rec). Data are presented in log2 (fold change) as mean ± SE, where *n* = 3 plants per treatment. Statistically significant differences were determined using student’s t-test and are shown as asterisks (* *p* < 0.05, ** *p* < 0.01, and *** *p* < 0.001)

In young leaves, the expression of *QsTIP2;1* increased significantly upon WS, while in the other tissues studied it either did not change or was significantly downregulated (roots and mature stems) (Fig. 9A). Conversely, upon rewatering, *QsTIP2;1* expression increased in young stems and decreased in leaves. The expression of *QsPIP2;4* followed a clearly different pattern upon stress, with significant upregulation in leaves and young stems and downregulation in roots and mature stems (Fig. 9B). Upon recovery, this pattern was kept in young stems, while in leaves, *QsPIP2;4* was downregulated, and in roots and mature stems it was kept similar to WW levels. Regarding *QsNIP1;2*, its expression was unaffected by the maturity of the tissue studied and was upregulated in leaves and downregulated in the other tissues upon WS while, after recovery, it was upregulated in roots and young stems (Fig. 9C). In contrast, *QsNIP6;1* transcript abundance remained below the detection limit under optimized RT-qPCR conditions used for the other *AQPs* of this study. Due to the lack of reproducible amplification across the biological replicates, *QsNIP6;1* was excluded from the final expression analysis.

## Discussion

### Phylogeny and functional diversity of cork oak AQPs

A detailed phylogenetic classification of *Q. suber* AQPs reveals their division into five well-known plant AQPs subfamilies: PIPs, TIPs, NIPs, SIPs, and XIPs, which is consistent with the AQPs in model higher plants, such as *Arabidopsis thaliana* and *Vitis vinifera* (Hussain et al. 2020; Sabir et al. 2021). The largest subfamily of PIPs is consistent with earlier reports suggesting that PIPs usually represent the most abundant group of AQPs involved in water transport across the plasma membrane in a wide range of plant species (Chaumont et al. 2000). Cork oak AQPs showed high sequence similarity to other species within the *Quercus* genus and Fagaceae family. The high degree of AQPs’ similarity is consistent with shared environmental pressures, such as drought and nutrient limitation and may reflect common molecular strategies for water management and stress adaptation in long-lived species (Berry et al. 2021).

The clear separation of cork oak aquaporins into these subfamilies suggests an evolutionary conservation of aquaporins for their essential roles in regulating water movement, nutrient uptake, and cellular turgor pressure. To explore this functional diversity, we selected a representative set of four AQPs (QsPIP2;4, QsTIP2;1, QsNIP1;2, and QsNIP6;1) for their detailed functional analysis. Among the selected proteins, QsPIP2;4 and QsTIP2;1 represent subfamilies generally associated with water transport, whereas QsNIP1;2 and QsNIP6;1, respectively from NIP-I and NIP-II subgroups, were included to assess broader substrate selectivity, including glycerol and metalloids, as described in other woody plants such as *Vitis vinifera* (Sabir et al. 2021). This selection was designed to represent major phylogenetic groups with distinct predicted transport properties and to examine whether phylogenetic classification is reflected in functional behavior. 

### Functional insights from transport assays

In the present study, the water and glycerol transport activities of cork oak AQPs were measured using stopped-flow spectroscopy, revealing significant variations among AQPs’ isoforms. This technique allows the precise measurement of water and glycerol movement across the intact yeast cell membrane, providing valuable insights into the functions of various AQPs’ types (Soveral et al. 2007; Sabir et al. 2020b).

QsPIP2;4 exhibited moderate water transport activity, whereas a Thr70Ser substitution in Clone B led to a loss of function. This finding suggests that minor variations in protein structure can affect AQPs’ functionality (Törnroth-Horsefield et al. 2006). However, the precise structural or regulatory basis of this effect requires further study. QsTIP2;1 exhibited the highest water permeability coefficient (*P*_*f*_*)* among the cloned AQPs, demonstrating a fourfold increase compared to the control strain. This finding is consistent with previous studies in which TIP AQPs expressed in yeast (Sabir et al. 2014) and in *Xenopus* oocytes (Li et al. 2008) showed higher water transport. This remarkable increase in water permeability in a heterologous system is in line with the efficient water transport capacity reported for TIPs. Moreover, overexpression of wheat TIP2;2 in *Arabidopsis* has been shown to enhance tolerance to drought and salinity, further supporting the relevance of TIPs in plant stress responses. By enabling cells to utilize vacuolar space, TIPs help maintain cellular integrity under osmotic stress, thereby contributing to resilience against environmental fluctuations (Kjellbom et al. 1999).

Furthermore, the glycerol transport activity exhibited by cloned QsNIPs reveals the functional diversity of NIP AQPs in cork oak. QsNIP1;2 demonstrated significantly high permeabilities to both water and glycerol, while QsNIP6;1 showed only glycerol transport. These differences agree with the subdivision of NIPs into groups with distinct substrate preferences. Their substrate specificity varies among their classified subgroups NIP-I, NIP-II, and NIP-III (Fig. 1) (Pommerrenig et al. 2015). Members of the NIP-I group (QsNIP1;2) showed the ability to transport both glycerol and water, while the NIP-II group members (QsNIP6;1) are majorly associated with glycerol transport (Wallace and Roberts 2005). Variability in substrate specificity among NIP isoforms has been linked to differences in the hydrophilic or hydrophobic nature of the ar/R residues of the pores (Sui et al. , 2001). The, The higher glycerol permeability of QsNIP1;2 as compared to QsNIP6;1 is consistent with the findings in our previous studies in grapevine NIPs (Sabir et al. 2020b).

After characterizing the transport properties of the selected aquaporins, their regulation was further examined under different pH conditions and in the presence of an inhibitor.

### AQPs gating by mercury chloride and pH regulation

The permeability of most cloned cork oak AQPs to water and glycerol was reduced in the presence of mercury chloride, consistent with its known role as an AQP inhibitor (Maurel et al. 2015). This inhibition typically occurs through mercury binding to conserved cysteine residues, causing conformational changes that block the pore and reduce the passage of water and small solutes like glycerol (Savage and Stroud 2007; Törnroth-Horsefield et al. 2006). While all cloned AQPs were evaluated for the expected inhibition of water and glycerol transport by mercury chloride, QsPIP2;4A was selected for further mechanistic investigation due to its unique activation by mercury chloride. This unusual response prompted an in-depth study to understand the underlying mechanisms. The activation was dose-dependent and was reversible with β-mercaptoethanol, similar to what has been reported for spinach SoPIP2;1 (Frick et al. 2013a) and *Medicago truncatula* (MtAQP1) (Krajinski et al. 2000), where mercury chloride increased water transport rather than inhibiting it. Before that, only rat AQP6 activation by mercury chloride was demonstrated (Yasui et al. 1999). The contrasting responses of these AQPs indicate the complexity of the gating mechanisms. It has been proposed that mercury activation may stabilize an open AQP conformation (Frick et al. 2013a; Kirscht et al. 2016; Xie et al. 2022). Although this activation is probably not involved in any physiological function, understanding the molecular basis for these conformational shifts could provide insights into AQPs’ gating mechanisms. Both QsPIP2;4 and SoPIP2;1 belong to the PIP2 subgroup of AQPs, sharing highly conserved sequences. PIPs generally have high sequence conservation, with a highly conserved cysteine in loop A that is thought to stabilize inter-monomeric bonds in PIP tetramers and form disulfide bonds with mercury chloride (Törnroth-Horsefield et al. 2006; Bienert et al. 2012). Both SoPIP2;1 and QsPIP2;4 possess Cys69 in loop A. Mutation of Cys69 to alanine (C69A) in QsPIP2;4 led to higher permeability than the wild-type, similar to observations in SoPIP2;1 (Kirscht et al. 2016). This increased permeability was speculated to be due to destabilization of the dimer and release of loop A, causing relaxation at the selectivity filter and tetrameric central pore opening, or indirectly affecting the monomeric pore structure (Kirscht et al. 2016). However, unlike the SoPIP2;1 mutant, the QsPIP2;4 mutant was unaffected by mercury chloride, suggesting that the mutation preventing the disulfide bridge leads to mercury insensitivity. It is important to note that some PIPs, such as AtPIP2;1, lack mercury sensitivity despite possessing the conserved cysteine (Verdoucq et al. 2008). Additionally, other AQP groups like TIPs and NIPs are mercury-sensitive without this conserved cysteine. This observation suggests that attributing the role of cysteine alone in mercury sensitivity may be an oversimplified approach and that other factors such as membrane physical properties may also be important. Changes in membrane fluidity due to mercury binding and minor conformational changes from mechanical stimuli cannot be excluded. Mechanosensitive gating has been observed in microbial, plant, and animal AQPs (Soveral et al. 1997; Wan et al. 2004; Fischer et al. 2009; Leitao et al. 2012; Tong et al. 2012).

Additionally, a pH-dependent gating was observed in cork oak AQPs. Intracellular pH (pH_in_) plays a crucial role in regulating their gating by reducing the water and glycerol permeabilities of QsPIP2;4, QsNIP1;2 and QsNIP6;1 upon cytosolic acidification, which is in accordance with previous studies (Tournaire-Roux et al. 2003). This pH-dependent regulation is mainly attributed to the histidine residue in loop D of PIPs (Törnroth-Horsefield et al. 2006; Frick et al. 2013b) and most of the TIPs (Leitao et al. 2012). The crystal structure of SoPIP2;1 demonstrates that upon cytosolic acidification, His gets protonated and changes the AQP conformation from open to closed (Törnroth-Horsefield et al. 2006; Frick et al. 2013b). This pH-dependent gating mechanism is thought to be a rapid response to environmental stresses like anoxic conditions during flood, allowing plants to control water flow across membranes (Tournaire-Roux et al. 2003). The high conservation of histidine residues was observed in all analyzed PIPs, supporting a common pH-sensing mechanism across different plant species.

However, QsTIP2;1 showed insensitivity to reduced intracellular pH. Similarly, a lack of pH sensitivity under various pH conditions was also observed in *Vvi*TIP1;1 (Shelden et al. 2009), while AtTIP5;1 gating was regulated by extracellular pH (Soto et al. 2010). Differential pH sensitivity of TIPs can be explained by variability in TIPs’ histidine conservation in the WebLogo presented in this study, indicating the involvement of the other key residues. Also, NIPs lack conservation of the histidine residue yet remain sensitive towards intracellular pH (Sabir et al. 2020b), emphasizing additional pH-dependent gating mechanisms that may be subfamily specific.

### Substrate sensitivity and detoxification role of cork oak AQPs

Beyond water and glycerol transport, the selected AQPs were further assessed for their ability to transport other atypical substrates. The drop-test growth assay conducted on cloned cork oak AQPs provided insights into the substrate specificity and potential physiological roles of various AQP isoforms. In this study, PIPs and TIPs showed no significant sensitivity to externally supplied substrates, suggesting that these AQPs are primarily involved in water homeostasis instead of the transport of other small solutes. A slight sensitivity to boron was observed, though less pronounced than in QsNIP6;1. It demonstrated apparent sensitivity to atypical substrates like arsenite, hydrogen peroxide, and boron, resulting in inhibited growth in their presence. This sensitivity indicates that QsNIP6;1 has broader substrate specificity than PIPs and TIPs. Its sensitivity to arsenite suggests that QsNIP6;1 may be involved in the detoxification of toxic metalloids, potentially functioning similarly to other NIP-type AQPs that transport small, neutral solutes, including metalloids such as boron and arsenite (Bienert et al. 2008). *Arabidopsis thaliana* NIP1;1 and NIP5;1 were suggested to transport arsenite and boron, respectively (Takano et al. 2006; Kamiya et al. 2009). Also, sensitivity to hydrogen peroxide aligns with previous findings that some NIPs facilitate the transport of reactive oxygen species (ROS), byproducts of environmental stress that can cause cellular damage (Bienert et al. 2008).

Overall, the yeast-based assays characterized the intrinsic transport properties of selected cork oak AQPs and provided the framework for subsequent analysis at the plant level.

### Immunolocalization of AQPs in cork oak stem tissue

The immunolocalization of TIP2s and PIP2s AQPs in young cork oak stems showed their distribution in the different cellular layers of the stem. It was specifically targeted to the PIP and TIP subfamilies using subgroup-specific antibodies, which can detect several members of the TIP2 and PIP2 subgroups. These AQPs represent the most abundant and primary contributors to cellular water transport in vascular tissues (Eisenbarth and Weig 2005; Gomes et al. 2009; Maurel et al. 2015; Shivaraj et al. 2021). In contrast, the NIP subfamily typically exhibits lower expression levels and more specialized, isoform-specific localization (Chevalier et al. 2015; Di Giorgio et al. 2016 ; Zhang et al. 2022) and its localization studies often rely on reporter-tagged constructs or specialized antibodies (Guenther et al. 2003; Wang et al. 2017; Beamer ZG et al. 2021) rather than broadly applicable, group-specific antibodies.

The predominant localization of TIP2 and PIP2 in the cortex, phelloderm, and phellogen layers was observed in the cork oak stem, suggesting their role in radial water transport across the stem. Similarly, in maize roots, an abundance of PIP2s was also observed in the cortex layer, facilitating water transport (Hachez et al. 2006). The phellogen, or cork cambium, is responsible for producing cork cells outwardly and phelloderm cells inwardly (Evert 2006). The presence of PIP2 AQPs in this meristematic layer and cortex is consistent with their potential role in providing water for cell division and expansion during cork formation and suberization. A high accumulation of PIP2s in maize roots was also observed, indicating their role in water uptake during the endodermis suberization (Hachez et al. 2006).

TIP2 and PIP2 were more evident in cells that did not undergo programmed cell death (PCD), while moving to outer layers TIP2 and PIP2 signals became weaker eventually disappearing completely from the outmost older layers of the cork. The absence of these signals in the outmost layers of cork cells can be explained by the fact that during maturation cork cells undergo suberization and PCD resulting in the formation of a highly dense tissue layer, which acts as a barrier, protecting the plant from water loss and environmental stresses (Pereira 2011). Different stages of PCD in cork cells were identified by (Inácio et al. 2021), and TIP2 AQPs were localized in the intact vacuolar membrane of the young cork cells. While moving outwardly no cytoplasm was observed and the cell lumen was filled with electron-dense material, thereby lacking TIP2 signals, suggesting that tonoplast rupture is occurring in the final stages of cell death (Inácio et al. 2021).

The subcellular localization of PIP2 and TIP2 revealed a stronger signal intensity for PIP2 compared to TIP2, suggesting a higher abundance of PIP2 AQPs in these tissues, consistent with prior findings that PIPs are the most abundant AQPs in plants and are primarily involved in facilitating water transport across the cell membrane (Maurel et al. 2015). The accumulation of PIP2 in the plasma membrane and around the nucleus (likely in the ER) is in accordance with the known trafficking pathway of these proteins (Chevalier and Chaumont 2015). Besides, it was also localized at structures similar to the membrane vesicles (Fig. 7I), which may require further ultrastructure studies. Moreover, given the broader substrate specificity of TIPs as compared to PIPs, their potential roles are beyond water transport. Furthermore, different vacuole types express specific TIP isoforms, highlighting their distinct functional roles within cellular processes (Wudick et al. 2009).

### Expression of cork oak AQPs during drought stress

AQPs of the PIP2 subfamily are considered as efficient water channels and some are relevant pathways for cell-to-cell water transport in plants (Kaldenhoff and Fischer 2006). With some exceptions, *PIP* genes are reported to be downregulated in the drought stress response in leaves (Afzal et al. 2016; Miranda et al. 2022). In contrast, we observed the upregulation of *QsPIP2;4* in cork oak leaves, similar to some accessions of drought-stressed *Arabidopsis* leaves (Alexandersson et al. 2010). In fact, in cork oak leaves, all three *AQPs* (*QsPIP2;4*, *QsTIP2;1* and *QsNIP1;2*) showed similar patterns, with upregulation at 10 days of WS, and downregulation (or no changes) after recovery (when compared to WW).

In roots, long-term exposure to water stress leads to lower AQPs’ activity to prevent reverse water transport from root cells to the water-depleted soil (Maurel et al. 2015; Afzal et al. 2016), while early drought conditions can trigger AQPs’ activity and expression to maximize the capture of available soil water and respond to water-limited conditions (Shekoofa and Sinclair 2018). Accordingly, all three cork oak *AQPs* studied were significantly downregulated after 10 days of irrigation at 10% Etc, and *QsNIP1;2* regained higher expression levels after the stress was over. Furthermore, all three *AQPs* (*QsPIP2;4*, *QsTIP2;1* and *QsNIP1;2*) were significantly upregulated in young stems during the recovery phase, which may reflect reacquiring normal water transport after a stress event. The upregulation is in line with the relevant role of AQPs during recovery from stress, namely in xylem parenchyma cells to help restore xylem hydraulic conductivity (Secchi et al. 2017). On the other hand, the absence of detectable *QsNIP6;1* transcript suggests that this AQP isoform may be expressed at extremely low levels or in a highly tissue- or condition-specific manner, which requires further investigation into its transcriptional regulation.

Notably, gas-exchange parameters showed only slight differences between WW and WS plants, suggesting that the imposed water stress may have been relatively mild under the tested conditions. This may indicate that either the applied stress duration was not sufficient or the inherent drought tolerance of cork oak (Besson et al. 2014) could not induce pronounced physiological changes. At the same time, changes in AQP expression were detected, which have been suggested as a highly sensitive early response to water deficit, even within a few hours of different stress implications (Pou et al. 2016; Suslov et al. 2024). Recently, *PIP2;1* in grapevine was shown to contribute to early stomatal closure while leaf hydraulic conductance (*K*_leaf_) was not changed (Albuquerque et al. 2026). Thus, the AQPs’ expression changes we observed may represent an early transcriptional adjustment to ongoing water limitation in cork oak, while a more pronounced physiological response may require a longer stress period.

## Conclusion

This study provides a comprehensive characterization of cork oak aquaporins by combining phylogenetic analysis, yeast-based functional assays, in situ immunolocalization, and drought-responsive expression profiling. The results indicate that PIP, TIP, and NIP subfamilies have distinct roles in water and solute transport. Together, these findings improve our understanding of aquaporin-mediated water relations in cork oak and offer a framework for future studies on their regulation under drought conditions. Future work on key residues of cork oak AQPs may help to clarify the mechanisms underlying gating and substrate selectivity. The unusual activation of QsPIP2;4 by mercury chloride also requires structural studies to clarify the molecular interactions involved, particularly how mercury binding stabilizes open conformations in PIP AQPs. Further investigation to confirm the AQPs’ trafficking route and their presence at various cellular structures needs ultrastructure studies to determine their contribution to cellular water dynamics and broader physiological processes. Additionally, exploring the genetic diversity of AQPs across cork oak populations may also provide insight into adaptive traits under varying climatic conditions. Taken together, these findings may provide applied approaches such as genetic engineering and precision forest management, helping to improve drought resilience in cork oak woodlands, offering sustainable solutions to mitigate the impacts of climate change and water scarcity on forest ecosystems.

## Supplementary Information

Below is the link to the electronic supplementary material.

Supplementary file1 (DOCX 19 KB)

Supplementary file2 (PDF 156 KB)

## Data Availability

All relevant data are provided within the article and the accompanying supporting information. The obtained sequence of the cloned QsPIP2;4 in this study has been submitted to the GenBank database (accession number [PV007186](https:/www.ncbi.nlm.nih.gov/nuccore/PV007186.1)).
