# Supplementary material for: Cork oak aquaporins: functional diversity and regulation with insights into drought response
Source: Plant Cell Rep. 2026 Jun 6;45(7):186. doi: 10.1007/s00299-026-03869-8 (PMC13242448; doi:10.1007/s00299-026-03869-8)
Supplement: Supplementary file 1 — Supplementary file1 (DOCX 19 KB) [file 299_2026_3869_MOESM1_ESM.docx]

Table S1. Primer sequences used in this study

| *Quercus suber* aquaporins | NCBI accession number | Primer name | Primer sequence (5´-3´) |
| --- | --- | --- | --- |
| QsPIP2;4 | PV007186 | Forward | GGTAGCTCTAGAATGGCTGCCAAGGATATTGAAGGGTTC |
|  |  | Reverse | ATACGCGTCGACCATGGCTGAAGAGCTCCTGAAGGAC |
| QsTIP2;1 | XP_023903922 | Forward | GGTAGCTCTAGAATGGCCAGGATTGCCTTTGGTC |
|  |  | Reverse | ATACGCGTCGACATACTCATTGGACAGAGGTGCATGTTC |
| QsNIP1;2 | XP_023887057 | Forward | GGTAGCTCTAGAATGGCTGGGAATTTAGGAACCAATGGC |
|  |  | Reverse | ATACGCGTCGACGATGGTGTTGTTGTTTTTTGACTCCTTTAGGAAAGAGC |
| QsNIP6;1 | XP_023898025 | Forward | GGTAGCTCTAGAATGGAAAACGAGGAAGTTCCATCAGCAC |
|  |  | Reverse | ATACGCGTCGACCCTTCTGAAGCTCCTTCTTGTTGAAGG |
| QsPIP2;4_C69A | -- | Forward | CATTCTACTGATCCG**GCT**ACTGGTGTTGGAATTCTTGGC |
|  |  | Reverse | GCCAAGAATTCCAACACCAGT**AGC**CGGATCAGTAGAATG |
| qPCR_QsPIP2;4 | -- | Forward | ATCTCAGGTGGGCACATTAAC |
|  |  | Reverse | TGCTGTATCCATCATTGAGCC |
| qPCR_QsTIP2;1 | -- | Forward | AGGCTTGGCAATTCCCATC |
|  |  | Reverse | TTGATCCACCGGAGAATGG |
| qPCR_QsNIP1;2 | -- | Forward | ATGCAGAAGTTGATTGCAGAG |
|  |  | Reverse | TGCTGGGTTAAAATGAGCAC |
| qPCR_QsNIP16;1 | Forward AAGGTGGGAGCTGAATTCG  Reverse ATGGTAACAGCTGGGTTGA | | |
| qPCR_QsGAPDH | -- | Forward | ACCGACTTCATTGGTGACAG |
|  |  | Reverse | AGATGCGATGTGGACAATCA |

* Restriction enzyme sequences are underlined. Base pairs for single amino acid substitution are bold and grey highlighted.

| *Quercus suber* aquaporins | Accession number | Length | Highest similarity with other plants BLASTP (%) |
| --- | --- | --- | --- |
| QsPIP2;4 | PV007186 | 281 | *Castanea mollissima* (98) |
| QsTIP2;1 | XP_023903922 | 248 | *Castanea mollissima* (99.6)  *Juglans regia* (96) |
| QsNIP1;2 | XP_023887057 | 277 | *Castanea mollissima* (92) |
| QsNIP6;1 | XP_023898025 | 304 | *Castanea mollissima* (97)  *Corylus avellana* (92) |

Table S2. Protein sequences similarity of cloned QsAQPs with other plants AQPs
