# Supplementary material for: Cork oak aquaporins: functional diversity and regulation with insights into drought response
Source: Plant Cell Rep. 2026 Jun 6;45(7):186. doi: 10.1007/s00299-026-03869-8 (PMC13242448; doi:10.1007/s00299-026-03869-8)
Supplement: Supplementary file 2 — Supplementary file2 (PDF 156 KB) [file 299_2026_3869_MOESM2_ESM.pdf]

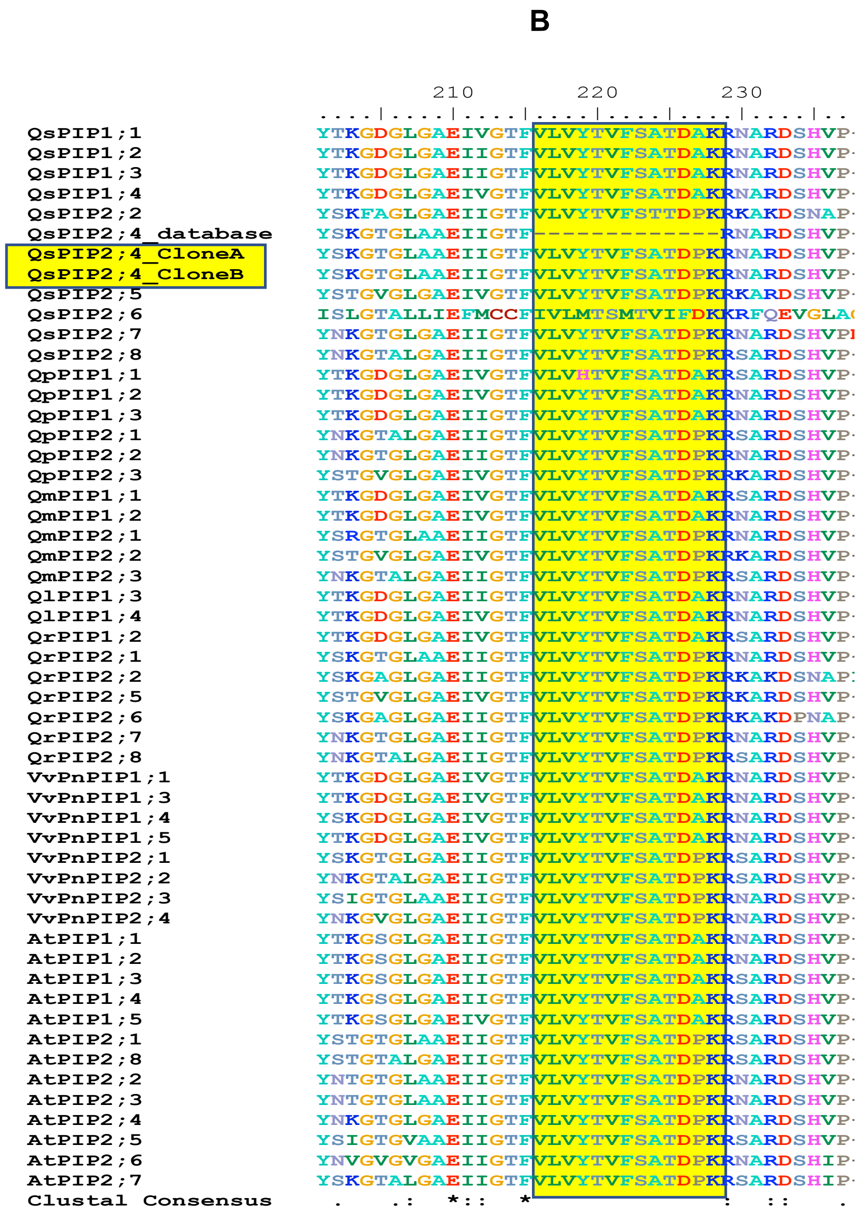


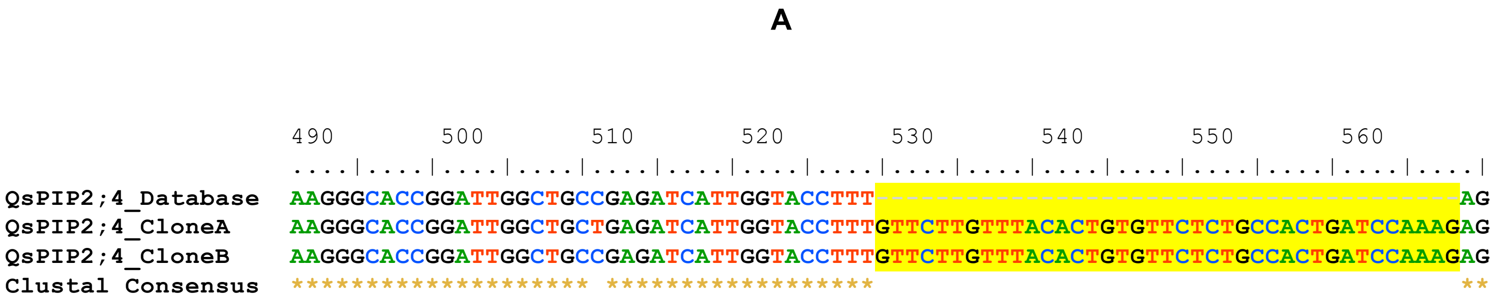


**Figure S1: Alignment of cork oak PIP2;4 sequences, comparing the reference database sequence with the sequences from two clones (A and B) obtained in this study. (A)**The nucleotide sequences alignment reveals a 39 bp nucleotide insertion in the cloned sequences. **(B)** The amino acid sequences also includes comparisons with PIP sequences from other *Quercus* species and reference plants (*Arabidopsis* and Vitis), revealing the missing 13 amino acids in the database sequence, leading to a shorter predicted protein.

```
                          70
                    .|....|....|...
QsPIP2;4_database   HSTDPCTGVGILGIA
QsPIP2;4_CloneA     HSTDPCTGVGILGIA
QsPIP2;4_CloneB     HSTDPCSGVGILGIA
Clustal Consensus   ****** ********
```

**Figure S2:** Alignment of two selected QsPIP2;4 transformants (Clone A and Clone B) with the database sequence reveals an amino acid substitution (Thr70Ser) in Clone B.
